# Safe Cooperative Decision-Making for Multi-UAV Pursuit–Evasion Games via Opponent Intent Inference

**DOI:** 10.3390/s26072243

**Published:** 2026-04-04

**Authors:** Wenxin Li, Yongxin Feng, Wenbo Zhang

**Affiliations:** School of Information Science and Engineering, Shenyang Ligong University, Shenyang 110158, China; liwenxin@sylu.edu.cn (W.L.); zhangwenbo@yeah.net (W.Z.)

**Keywords:** cooperative multi-UAV, pursuit–evasion, partial observability, intent inference, hierarchical reinforcement learning

## Abstract

Cooperative multi-UAV pursuit–evasion under occlusions and sensor noise is challenged by intermittent observability of the evader, varying observation-window lengths, and non-stationary evader tactics, all of which destabilize prediction and undermine safety-constrained cooperation. To address these challenges, we propose a safe decision-making framework that uses behavior mode and subgoal inference as intermediate representations for interpretable, uncertainty-aware cooperation. Specifically, an observation-driven generative intent–subgoal model infers the evader’s behavior mode and subgoal from short observation windows. Building on this model, a length-agnostic trajectory predictor is trained via multi-window knowledge distillation and consistency regularization to produce future trajectory predictions with calibrated uncertainty for arbitrary observation-window lengths, thereby reducing cross-window inference inconsistency and lowering online computational cost. Based on these predictions, we derive belief and risk features and develop a belief–risk-gated hierarchical multi-agent policy based on soft actor-critic with a safety projection layer, enabling adaptive strategy switching and a controllable trade-off between efficiency and safety. Experiments in obstacle-rich pursuit–evasion environments with randomized layouts and diverse obstacle configurations demonstrate more stable cooperative capture, safer maneuvering, and lower decision variance than representative baselines, indicating strong robustness and real-time feasibility. Specifically, across different observation-window settings, the proposed method improves the normalized expected return by approximately 5–7% over the strongest baseline and reduces pursuer losses by roughly 22–25%. Moreover, its end-to-end decision latency consistently remains within the 50 ms control cycle.

## 1. Introduction

Cooperative multi-UAV pursuit–evasion concerns adversarial decision-making in constrained airspace, where multiple pursuer UAVs must coordinate to track, contain, and intercept a continuously maneuvering evader. This setting arises in tasks such as perimeter protection of restricted areas, low-altitude security around critical infrastructure, and the handling of hazardous UAVs in disaster-response scenarios. In this context, safe cooperative decision-making refers not only to improving capture effectiveness but also to ensuring that the pursuer team remains operationally feasible throughout coordinated maneuvers by satisfying obstacle-avoidance, inter-UAV collision-avoidance, and communication-limited coordination constraints. Because the pursuers typically have access only to local and incomplete observations, they must also infer the evader’s current intent online. In this paper, opponent intent inference specifically denotes estimating the evader’s behavior mode and short-horizon spatial subgoal from short observation sequences, thereby providing structured semantic cues for downstream forecasting and cooperative decision-making.

Compared with cooperative pursuit–evasion on land or water, multi-UAV pursuit–evasion games involve much higher maneuvering speeds, faster closing dynamics, and stronger sensitivity to line-of-sight conditions. As a result, local occlusions, abrupt target appearance and disappearance, and communication constraints can more readily disrupt situational continuity and coordination consistency. At the same time, the problem studied here is fundamentally different from conventional UAV trajectory planning, formation control, or pure obstacle avoidance. Those tasks typically assume predefined mission objectives and comparatively passive environmental constraints, with the primary goal of generating feasible trajectories that satisfy dynamic and safety requirements. In contrast, in pursuit–evasion games, the evader actively maneuvers and switches strategies in response to the evolving interaction, creating a tightly coupled closed loop among perception, prediction, and decision-making.

In realistic environments, however, the evader is often only intermittently visible because of occlusions and sensor noise, and the available observation window may change over time. This makes short-term forecasting unstable and weakens end-to-end policy mappings that depend on a fixed observation pattern. The problem becomes harder when the evader changes its tactic during the interaction. Similar observations may then lead to different future motions, which increases decision uncertainty. Safety adds another layer of difficulty. Capture efficiency, obstacle avoidance, inter-UAV collision avoidance, and communication-limited coordination are tightly coupled during cooperative maneuvers. If predictive uncertainty is not represented explicitly and incorporated into decision-making, the policy cannot regulate this trade-off in a controlled manner.

Classical methods for cooperative pursuit–evasion are mainly built on differential games, reachability analysis, and geometric guidance laws. These methods model inter-agent interactions explicitly and can produce reliable strategies when accurate state information is available and repeated planning is affordable [1,2]. Their limitations become more evident under partial observability and limited communication. They often rely on frequent replanning and a reasonably consistent shared view of the situation. As the team grows or the environment becomes more complex, the associated computation and coordination costs can increase rapidly. Moreover, conservative safety handling may preserve feasibility but reduce pursuit effectiveness. Thus, although these methods address coordination and constraints, they do not fully resolve the combined need for interpretable opponent reasoning, stable prediction from incomplete observations, and real-time risk-aware cooperation.

Recent multi-agent learning methods reduce the dependence on accurate models and online search, and therefore offer stronger adaptability in complex environments [3,4,5]. Several studies have already considered uncertainty, partial observability, or safety constraints. Even so, three key gaps remain in the present setting. First, many methods still compress perception, prediction, and control into a single end-to-end mapping. This leaves the evader’s short-term behavioral semantics implicit, which makes the policy harder to interpret and less stable when visibility is intermittent or tactics change. Second, existing predictors often assume a fixed observation length, or they process variable-length histories without explicitly enforcing cross-length consistency. Their outputs may therefore become unstable when observation windows vary or measurements are missing. Third, current safe learning methods usually handle uncertainty through reward penalties, shielding rules, or action correction. In most cases, predictive uncertainty is not turned into an explicit decision variable that can regulate safety margins, trigger mode switching, and guide cooperation at the policy level. In other words, prior work has addressed uncertainty-aware learning and safety-aware control, but it has not fully closed the loop between interpretable opponent inference, variable-length online prediction, and uncertainty-driven cooperative decision-making in multi-UAV pursuit–evasion.

To address these challenges, we propose a multi-level closed-loop framework for cooperative multi-UAV pursuit–evasion under partial observability, in which opponent intent inference is explicitly coupled with safe cooperative decision-making. The framework integrates interpretable inference, real-time prediction, and risk-aware cooperation in a unified pipeline. First, it infers the evader’s behavior mode and subgoal from short observation windows, yielding structured intermediate representations for downstream decision-making. Second, these representations are distilled into a lightweight online predictor that delivers future trajectory forecasts together with calibrated uncertainty under missing observations and varying observation-window lengths. Finally, a belief–risk-gated hierarchical multi-agent policy incorporates belief and risk features derived from these predictions to regulate safety margins, switch strategy modes, and realize a controllable trade-off between efficiency and safety.

The main contributions of this work are summarized as follows:

- To address the lack of explicit intermediate semantics in cooperative pursuit–evasion under partial observability, we develop a structured inference framework that estimates the evader’s behavior mode and spatial subgoal from short observation windows. These variables provide interpretable and reusable representations for downstream prediction and cooperative decision-making.
- To address the instability of existing predictors under intermittent visibility and varying observation-window lengths, we propose a length-agnostic knowledge distillation paradigm. It transfers structured semantic knowledge from a generative teacher model to a lightweight online predictor, enabling more consistent forecasting together with calibrated uncertainty estimation for downstream risk assessment.
- To address the limited use of predictive uncertainty in existing safe multi-agent decision methods, we propose a belief–risk-gated hierarchical policy optimization method. This method converts predictive uncertainty into explicit risk features and uses them to regulate safety margins, switch policy modes, and support a controllable trade-off between pursuit efficiency and operational safety.
- We conduct comprehensive experiments under diverse information constraints and environmental variations. The results show that the proposed framework delivers stronger cooperative effectiveness, better decision stability, and more reliable safety performance than representative baselines, especially when visibility is intermittent, observation histories are variable in length, and the environment becomes more complex.

The remainder of this paper is organized as follows. Section 2 reviews related work on learning-based cooperative pursuit–evasion decision-making, inference under partial observability, and safety-constrained control. Section 3 presents the system model and problem formulation. Section 4 details the proposed hierarchical framework. Section 5 reports comparative and ablation studies under diverse information constraints and environmental variations.

## 2. Related Work

### 2.1. Learning-Based Cooperative Pursuit–Evasion Decision-Making

Learning-based cooperative pursuit–evasion has been widely studied in scenarios with coupled continuous dynamics, obstacle and collision avoidance, and multi-agent coordination, where policies must respond online to adversarial interactions. Recent deep reinforcement learning methods have made this line of research more adaptive and more suitable for real-time deployment. A representative direction combines pursuit–evasion planning with learned policies so that the system can preserve cooperative capture performance in known environments while adapting to unknown or changing conditions through online inference and policy updates [3,6]. Another direction emphasizes decentralized cooperation in heterogeneous or multi-target settings, where role assignment and complementary maneuvers are learned to improve encirclement efficiency and coordination stability [7,8].

As the team size increases and the constraints become more strongly coupled, scalability and feasibility become central concerns. Feasible maneuver learning with collision-free guarantees has been developed to preserve cooperative stability in larger teams [9]. Under restricted fields of view or communication limits, missing-information modeling and coordination-aware objectives have been incorporated to strengthen decentralized information fusion and formation maintenance [10,11,12]. To reduce interaction cost and improve sample efficiency, offline reinforcement learning and related paradigms have also been introduced into pursuit–evasion problems in other unmanned domains, such as underwater systems [13]. From the perspective of control and optimization, distributed optimal solutions and capture or formation control provide theoretical support for the stability and convergence of cooperative maneuvers [14]. Policy reuse and cross-scenario transfer further reduce training and deployment costs by leveraging previously learned skills or policy libraries [15,16].

Taken together, these studies have improved online performance, coordination efficiency, and scalability. Their main focus, however, is still on learning effective actions rather than maintaining an explicitly interpretable opponent belief for decision regulation. In most cases, opponent modeling is absorbed into policy parameters, value functions, or auxiliary estimators. As a result, behavior mode and spatial subgoal are not maintained as continuously updated decision variables. This becomes a clear limitation when visibility is intermittent, observation histories vary in length, and the opponent changes tactics over time. In contrast, our method explicitly represents the evader’s behavior mode and spatial subgoal as intermediate variables and uses them to support downstream prediction and cooperative decision-making.

### 2.2. Cooperative Pursuit–Evasion Under Partial Observability

Occlusions, limited fields of view, and intermittent communication naturally introduce partial observability into cooperative pursuit–evasion. They also lead to delayed, inconsistent, or missing information. Existing studies have mainly addressed this issue from three directions. The first direction encodes missing information and coordination cost directly into policy inputs or learning objectives so that decentralized cooperation remains effective even when local observations are incomplete [10]. The second direction introduces observation compensation, state estimation, or uncertainty observers to handle unknown disturbances and local observation bias, thereby improving robustness under partial observability [17]. Related challenges have also been studied in other networked autonomous systems. For example, event-based $H_{\infty }$ filtering has been developed for networked mass-switching autonomous marine vehicles to improve state estimation under external disturbances, bandwidth limitations, and transmission delays [18]. The third direction incorporates occlusion and visibility constraints into decision-making itself, encouraging actions that preserve or enlarge visible regions in order to improve information quality and capture robustness [19,20]. In addition, pre-trained policies and policy libraries have been explored to accelerate initialization and reduce solution cost in large-scale adversarial settings [15]. Beyond observation incompleteness itself, practical multi-UAV collaboration also depends on secure and efficient information exchange over imperfect networks. Comparative studies of QUIC/UDP and TLS/TCP in unsafe networks have revealed nontrivial trade-offs among delay, packet loss, resource usage, and attack resilience [21]. Although such studies do not address opponent reasoning or cooperative policy learning under partial observability, they further highlight the importance of communication quality for timely and reliable information sharing in cooperative systems.

These efforts have substantially improved cooperation under incomplete information, but their main goal is usually to recover missing state information, reduce observation bias, or preserve visibility. In other words, uncertainty is still treated primarily as a state-estimation or observation-quality problem. Much less attention has been given to high-level behavioral uncertainty induced by local observation sequences, especially when the target appears intermittently, the available history length changes over time, and the opponent’s strategy is non-stationary. Under such conditions, existing methods generally do not maintain a generative and length-adaptive belief over behavior mode and spatial subgoal. They also rarely transform predictive uncertainty into explicit variables for risk assessment, tactical switching, and hierarchical control. Our method differs from this line of work in that it does not stop at state completion or visibility preservation. Instead, it builds a structured belief from local observation sequences, keeps prediction consistent under variable-length observations, and then feeds uncertainty-aware predictions into downstream cooperative decision-making.

### 2.3. Safety-Constrained Cooperative Pursuit–Evasion Decision-Making

Safety in pursuit–evasion is tightly coupled with task efficiency because obstacle avoidance, inter-UAV collision avoidance, input bounds, kinematic limits, and closed-loop feasibility all directly affect cooperative capture performance. To address these issues, prior studies have explored hierarchical reinforcement learning, decentralized inference, and constraint-aware control. Hierarchical reinforcement learning and decentralized inference mechanisms have been used to handle temporal coupling, delayed updates, and cooperative stability in constraint-intensive settings [22,23]. Environmental structure has also been exploited to support feasible cooperation and to improve encirclement efficiency in cluttered obstacle fields [24]. In group-adversarial settings with stronger uncertainty, hierarchical modeling and uncertainty-driven training mechanisms have been introduced to enhance robustness [25], while preserving feasibility under collision-avoidance coupling remains a persistent challenge in large-scale cooperation [9].

In parallel, safe reinforcement learning has increasingly been combined with explicit constraint-enforcement tools. Control barrier functions embed safe sets into policy execution through action correction and therefore provide structured constraint satisfaction for learning-based pursuit–evasion [26]. Recovery mechanisms and multi-step safety checks have also been studied to reduce the probability of violations in high-speed or high-risk interactions [27]. Observer-driven multi-agent learning further improves adaptability to unknown disturbances and information mismatch [17].

These studies have advanced safety and closed-loop feasibility in a meaningful way. Still, most existing approaches handle safety through external constraint modules, recovery layers, robustness enhancement, or action-level correction. Safety is therefore often imposed on the policy at the execution level, rather than entering the cooperative decision process as a high-level variable coupled with opponent belief. This design can improve feasibility, but it may also lead to conservative behavior, limited control over risk allocation, and weak support for tactical mode switching in long-horizon pursuit–evasion. This limitation becomes more pronounced in occlusion-rich environments with non-stationary opponent maneuvers. Our method differs in that predictive uncertainty over behavior mode and spatial subgoal is explicitly converted into risk features, which are then used for risk assessment, policy-mode switching, and safety-margin regulation in a closed loop.

## 3. System Model and Problem Formulation

### 3.1. System Model

We consider a multi-UAV pursuit–evasion game in a planar workspace and model pursuer-team decision-making as a state-augmented decentralized partially observable Markov decision process (Dec-POMDP), in which the environment state is augmented with compact latent variables that encode the evader’s behavior mode and subgoal.

Let $N^{f}=\left\{1,\ldots ,N_{f}\right\}$ and $N^{a}=\left\{1,\ldots ,N_{a}\right\}$ denote the pursuer and evader index sets, respectively. The formulation below is general, while the evaluation simulations in Section 5 use the 2:2 setting $N_{f}=N_{a}=2$. For any UAV $k\in (N^{f}\cup N^{a})$, define

(1) $$x_{k}\left(t\right)\;=\;\left[\begin{array}{c} p_{k}\left(t\right)\\ v_{k}\left(t\right) \end{array}\right]\in R^{4},p_{k}\left(t\right)=\left[\begin{array}{c} p_{k,x}\left(t\right)\\ p_{k,y}\left(t\right) \end{array}\right],v_{k}\left(t\right)=\left[\begin{array}{c} v_{k}^{x}\left(t\right)\\ v_{k}^{y}\left(t\right) \end{array}\right],$$

where $p_{k,x}\left(t\right)$ and $p_{k,y}\left(t\right)$ denote the *x*- and *y*-coordinates of $p_{k}\left(t\right)$. Under planar acceleration control

(2) $$u_{k}\left(t\right)\;=\;a_{k}\left(t\right)\;=\;\left[\begin{array}{c} a_{k}^{x}\left(t\right)\\ a_{k}^{y}\left(t\right) \end{array}\right]\in R^{2},$$

the kinematics are given by

(3) $${\dot{x}}_{k}\left(t\right)\;=\;f\left(x_{k}\left(t\right),u_{k}\left(t\right)\right)\;=\;\left[\begin{array}{c} v_{k}\left(t\right)\\ a_{k}\left(t\right) \end{array}\right],$$

subject to

(4) $$∥v_{k}\left(t\right){∥}_{2}\leq v_{k}^{\max},\;∥a_{k}\left(t\right){∥}_{2}\leq a_{k}^{\max}.$$

With sampling period Δt and piecewise-constant controls, let $x_{k}^{t}:=x_{k}\left(t\Delta t\right)$ and $u_{k}^{t}:=u_{k}\left(t\Delta t\right)$. First-order Euler discretization yields

(5) $$x_{k}^{t+1}\;=\;x_{k}^{t}+\Delta t\;f\left(x_{k}^{t},u_{k}^{t}\right)\;=\;\left[\begin{array}{c} p_{k}^{t}+\Delta t\;v_{k}^{t}\\ v_{k}^{t}+\Delta t\;a_{k}^{t} \end{array}\right],$$

and the same bounds are imposed on $v_{k}^{t}$ and $a_{k}^{t}$. Denote the global physical state by $X_{t}=\left\{x_{k}^{t}:k\in N^{f}\cup N^{a}\right\}.$

Pursuers communicate over a time-varying graph $G_{t}^{\mathrm{comm}}=\left(N^{f},E_{t}^{\mathrm{comm}}\right)$ with link indicator $c_{i\ell }^{t}\in \left\{0,1\right\}$:

(6) $$\left(i,\ell \right)\in E_{t}^{\mathrm{comm}}⟺c_{i\ell }^{t}=1,\;i,\ell \in N^{f},\;i\neq \ell ,$$

and neighbor set

(7) $$N_{i}^{\mathrm{comm}}\left(t\right)\;=\;\left\{\ell \in N^{f}\setminus \left\{i\right\}\;|\;c_{i\ell }^{t}=1\right\}.$$

Communication links are treated as bidirectional, i.e., $c_{i\ell }^{t}=c_{\ell i}^{t}$, and $G_{t}^{\mathrm{comm}}$ is updated at each discrete time step. When $c_{i\ell }^{t}=1$, pursuers *i* and *ℓ* exchange their current self-states before action selection at time *t*, so that neighbor states can be incorporated into $o_{i}^{t}$; otherwise, no information from that agent is available at step *t*.

Let $N_{i}^{\mathrm{vis}}\left(t\right)=\left\{j\in N^{a}|p_{j}^{t}\in V_{i}^{t}\right\}$ denote the set of visible evaders, where $V_{i}^{t}$ is the occlusion-aware field of view defined in (15). Given $G_{t}^{\mathrm{comm}}$ and $N_{i}^{\mathrm{vis}}\left(t\right)$, pursuer *i* forms

(8) $$o_{i}^{t}\;=\;\left[x_{i}^{t},\;{\left\{x_{\ell }^{t}\right\}}_{\ell \in N_{i}^{\mathrm{comm}}\left(t\right)},\;{\left\{{\tilde{p}}_{i,j}^{t},{\tilde{v}}_{i,j}^{t}\right\}}_{j\in N_{i}^{\mathrm{vis}}\left(t\right)}\right].$$

Here, ${\tilde{p}}_{i,j}^{t}$ and ${\tilde{v}}_{i,j}^{t}$ denote the relative position and velocity, defined by ${\tilde{p}}_{i,j}^{t}:=p_{j}^{t}-p_{i}^{t}$ and ${\tilde{v}}_{i,j}^{t}:=v_{j}^{t}-v_{i}^{t}$.

To make the latent augmentation explicit, we use a discrete variable $z_{t}\in Ƶ$ to represent the evader’s behavior mode and a continuous variable $g_{t}\in G\subset R^{d_{g}}$ to represent its short-horizon subgoal. The pair $\eta_{t}=\left(z_{t},g_{t}\right)$ provides a compact description of high-level evader intent. The main symbols used in this subsection are summarized in Table 1 for ease of reference.

The cooperative decision-making problem of the pursuer team is abstracted as a state-augmented decentralized partially observable Markov decision process:

(9) $$M^{\mathrm{aug}}\;=\;\langle N^{f},\;S^{\mathrm{aug}},\;{\left\{A_{i}\right\}}_{i\in N^{f}},\;{\left\{O_{i}\right\}}_{i\in N^{f}},\;P^{\mathrm{aug}},\;O,\;r,\;\gamma \rangle .$$

Here, $A_{i}$ is the action space of agent *i*, corresponding to the constrained acceleration control $u_{i}^{t}$; $O_{i}$ is its local observation space; and the discount factor satisfies γ∈(0,1]. The augmented state space is defined as

(10) $$S^{\mathrm{aug}}\;=\;X\times Ƶ\times G,$$

where X is the domain of the global physical state $X_{t}$, Ƶ is the discrete behavior-mode space, and G is the continuous subgoal space. Accordingly, the augmented state at time *t* is

(11) $$S_{t}\;=\;\left(X_{t},\;z_{t},\;g_{t}\right)\in S^{\mathrm{aug}}.$$

At the beginning of each episode, the latent state is initialized by a prior

(12) $$z_{0}∼p_{0}\left(z\right),\;g_{0}∼p_{0}\left(g|z_{0}\right),$$

where $p_{0}\left(z\right)$ is a categorical prior over behavior modes and $p_{0}\left(g|z_{0}\right)$ is a mode-conditioned Gaussian prior over subgoals. In practice, once the first valid observation window becomes available, this prior is replaced by an inferred belief from the observation sequence.

The latent variables evolve through an augmented transition kernel of the form

(13) $$P^{\mathrm{aug}}\;\left(S_{t+1}|S_{t},u_{t}^{f},u_{t}^{a}\right)=P\;\left(X_{t+1}|X_{t},u_{t}^{f},u_{t}^{a}\right)\;P_{z}\;\left(z_{t+1}|z_{t},X_{t}\right)\;P_{g}\;\left(g_{t+1}|z_{t+1},X_{t+1}^{a}\right),$$

where $X_{t+1}^{a}=\left\{x_{j}^{t+1}:j\in N^{a}\right\}$ denotes the evader-team state, $P_{z}$ models mode switching, and $P_{g}$ models the evolution of the short-horizon subgoal under the current mode. This factorization is used to define the augmented Dec-POMDP at the modeling level.

In implementation, however, the pursuers do not sample $z_{t}$ and $g_{t}$ directly from $P_{z}$ and $P_{g}$ during online decision-making. Instead, they maintain a belief over the latent variables and update it from the most recent observation window. Specifically, the latent belief at step *t* is inferred from $o_{i}^{t-L+1:t}$ by the learned inference model introduced later, so the practical latent distribution evolves through repeated amortized inference rather than explicit latent-state simulation.

In addition, $P^{\mathrm{aug}}$ denotes the augmented transition kernel, *O* denotes the observation kernel that characterizes how the local observation $o_{i}^{t}$ is generated from the augmented state under communication and visibility constraints, and $r(S_{t},u_{t}^{f},u_{t}^{a})$ is the per-step reward function, where $u_{t}^{f}\;=\;{\left\{u_{i}^{t}\right\}}_{i\in N^{f}},u_{t}^{a}\;=\;{\left\{u_{j}^{t}\right\}}_{j\in N^{a}}$ are the joint control inputs of the pursuer and evader teams, respectively.

### 3.2. Problem Formulation

**Figure 1:**
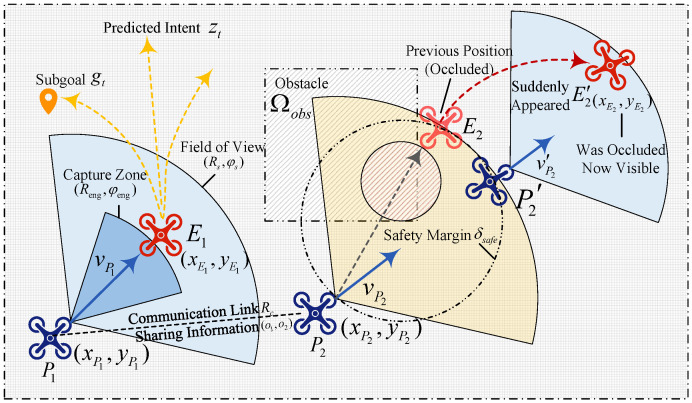
A 2:2 obstacle-rich pursuit–evasion setting. Pursuers communicate within radius $R_{c}$ under LoS, observe evaders in the occlusion-aware field of view $\left(R_{s},\varphi_{s}\right)$, and engage evaders in the capture cone $\left(R_{\mathrm{eng}},\varphi_{\mathrm{eng}}\right)$ with safety margin $\delta_{\mathrm{safe}}$. Obstacles induce intermittent observability by blocking LoS.

Let $\Omega \subset R^{2}$ be a bounded task space with static obstacles $\Omega_{\mathrm{obs}}\subset \Omega$ and free space $\Omega_{\mathrm{free}}=\Omega \setminus \Omega_{\mathrm{obs}}$, as shown in Figure 1. Obstacles induce both motion constraints and line-of-sight (LoS) occlusions. With pursuer communication radius $R_{c}$, $c_{i\ell }^{t}$ is defined as

(14) $$c_{i\ell }^{t}\;=\;I\left(∥p_{i}^{t}-p_{\ell }^{t}{∥}_{2}\leq R_{c},\;\mathrm{LOS}\left(p_{i}^{t},p_{\ell }^{t}\right)=1\right),$$

where LOS(·,·)∈{0,1} indicates whether the connecting segment intersects $\Omega_{\mathrm{obs}}$. This definition yields a geometry-induced, time-varying communication graph that accounts for obstacle occlusions: links may be broken either because the distance exceeds $R_{c}$ or because the LoS is blocked.

Each UAV has a limited occlusion-aware field of view with range $R_{s}$ and angle $\varphi_{s}$. For $∥v_{k}^{t}{∥}_{2}\neq 0$, define the heading $e_{k}^{t}=\frac{v_{k}^{t}}{∥v_{k}^{t}{∥}_{2}}$, and when $∥v_{k}^{t}{∥}_{2}=0$, $e_{k}^{t}$ is set to $e_{k}^{t-1}$ (initialized by the first nonzero-velocity heading) to keep the field of view well defined. Then

(15) $$\begin{array}{c} V_{k}^{t}=\{p\in \Omega_{\mathrm{free}}|\;∥p-p_{k}^{t}{∥}_{2}\leq R_{s},∠\;\left(p-p_{k}^{t},\;e_{k}^{t}\right)\leq \frac{\varphi_{s}}{2},\mathrm{LOS}\left(p_{k}^{t},p\right)=1\}. \end{array}$$

The evader policy is non-stationary and may switch between behavior modes. A discrete mode variable $z_{t}\in Ƶ$ is introduced, and the evader is modeled by mode-driven stochastic policies

(16) $$\Pi^{a}\;=\;\left\{\pi^{a}\;|\;u_{t}^{a}∼\pi^{a}\left(\;\cdot \;|X_{t},z_{t}\right),\;z_{t+1}∼P_{z}\left(\;\cdot \;|z_{t},X_{t}\right)\right\},$$

where $u_{t}^{a}:={\left\{u_{j}^{t}\right\}}_{j\in N^{a}}$ denotes the joint action of the evader team and $P_{z}$ is an unknown Markov kernel that captures situation-dependent mode switching. In particular, four interpretable evader behavior modes are considered, with $z_{t}\in \left\{0,1,2,3\right\}$. When $z_{t}=0$, the evader follows an escape mode that primarily increases its distance from the nearest pursuer. When $z_{t}=1$, the evader adopts an occlusion-seeking mode and tends to move along obstacle boundaries so as to reduce sustained observability. When $z_{t}=2$, the evader executes a deception mode by introducing rapid heading and velocity variations, thereby intentionally inducing multimodal futures under short observation windows. When $z_{t}=3$, the evader switches to a counter-engagement mode and actively exploits local advantages to challenge the nearest pursuer, thereby creating an escape opportunity for its teammate in the 2:2 setting.

Short observation windows often induce multimodal future-motion distributions. Let $h_{i}^{t}=\left(o_{i}^{0},\ldots ,o_{i}^{t}\right)$ denote the full observation history of pursuer *i*, and let $o_{i}^{t-L+1:t}$ denote the most recent window of length L∈N. Under occlusions and non-stationary maneuvers, similar short-window evidence may correspond to different future trajectories. Therefore, the conditional distribution $p(X_{t+1:t+H}^{a}|o_{i}^{t-L+1:t})$ is generally multimodal, where $X_{t+1:t+H}^{a}$ denotes the future trajectory of the evader team over a prediction horizon H∈N.

To capture this multimodality, we introduce a continuous subgoal variable $g_{t}\in G\subset R^{d_{g}}$. The variable $g_{t}$ summarizes the evader team’s short-horizon motion objective at time *t*, such as a mode-dependent waypoint for each evader over the near horizon. Conceptually, $g_{t}∼q\left(\cdot |o_{i}^{t-L+1:t}\right)$, where q(·∣·) denotes an observation-consistent posterior used for subgoal inference under partial observability.

To preserve the Markov property in the augmented-state formulation, we treat $\left(z_{t},g_{t}\right)$ as latent environment variables with unknown situation-dependent dynamics. The pursuers do not observe these variables directly. Instead, they infer them from observation histories. Accordingly, the augmented latent variable introduced in Section 3.1 is instantiated as $\eta_{t}=\left(z_{t},g_{t}\right)$, and the augmented state is written as $S_{t}=\left(X_{t},z_{t},g_{t}\right)$.

The latent variables are assumed to follow a Markov kernel:

(17) $$z_{t+1}∼P_{z}\left(\;\cdot \;|z_{t},X_{t}\right),\;g_{t+1}∼P_{g}\left(\;\cdot \;|z_{t+1},X_{t+1}^{a}\right),$$

where $P_{g}$ is an unknown conditional distribution that describes how the short-horizon subgoal evolves with the current mode and evader state. Under this convention, the augmented transition factorizes as

(18) $$\begin{array}{cc} P^{\mathrm{aug}}\left(S_{t+1}|S_{t},u_{t}^{f},u_{t}^{a}\right) & =P\left(X_{t+1}|X_{t},u_{t}^{f},u_{t}^{a}\right)\;P_{z}\left(z_{t+1}|z_{t},X_{t}\right)\times P_{g}\left(g_{t+1}|z_{t+1},X_{t+1}^{a}\right), \end{array}$$

This completes the state augmentation while leaving $P_{z}$ and $P_{g}$ unspecified for later learning-based approximation.

Engagement is defined using radius $R_{\mathrm{eng}}\leq R_{s}$ and view angle $\varphi_{\mathrm{eng}}\leq \varphi_{s}$. For an ordered cross-team pair (i,j), with relative velocity $v_{i,j}^{t}=v_{j}^{t}-v_{i}^{t}$, the engagement indicator is

(19) $$I_{i\to j}^{t}\;=\;\left\{\begin{array}{cc} 1, & \mathrm{if}\;∥p_{j}^{t}-p_{i}^{t}{∥}_{2}\;\leq \;R_{\mathrm{eng}},\;∠\;\left(p_{j}^{t}-p_{i}^{t},\;e_{i}^{t}\right)\leq \frac{\varphi_{\mathrm{eng}}}{2},\mathrm{LOS}\left(p_{i}^{t},p_{j}^{t}\right)=1,\;{\left(p_{j}^{t}-p_{i}^{t}\right)}^{⊤}v_{i,j}^{t}\leq 0;\\ 0, & \mathrm{otherwise}. \end{array}\right.$$

The last inequality enforces a closing condition, so the target is not moving away along the line of sight. Equation (19) applies to any ordered cross-team pair. Thus, *i* may denote either a pursuer or an evader, while *j* is selected from the opposite team. Given a dwell length $\tau_{\mathrm{eng}}\in N$, capture occurs if

(20) $$\sum_{s=t-\tau_{\mathrm{eng}}+1}^{t}I_{i\to j}^{s}\;\geq \;\tau_{\mathrm{eng}}.$$

Let $\sigma_{k}^{t}\in \left\{0,1\right\}$ denote the survival indicator for $k\in N^{f}\cup N^{a}$, where $\sigma_{k}^{t}=0$ indicates that the UAV is removed from the game and remains inactive thereafter. The number of surviving pursuers is denoted by $N_{\mathrm{surv}}^{t}=\sum_{k\in N^{f}}\sigma_{k}^{t}$. To make removal explicit, we apply the same dwell rule in both directions. An evader $j\in N^{a}$ is captured when there exists a pursuer $i\in N^{f}$ that satisfies (20). Symmetrically, a pursuer $i\in N^{f}$ is lost when there exists an evader $j\in N^{a}$ that satisfies the same condition with the roles exchanged. Once the dwell condition is met at time *t*, the corresponding survival indicator is set to zero from that step onward. The removed UAV then no longer participates in sensing, communication, or control.

Step rewards are built from physically interpretable quantities. They serve three purposes: they encourage capture by reducing pursuer–evader separation, preserve survivability and safety margins, and discourage excessive control effort. We first define distance-based indicators for opportunity and safety clearance. We then map them to smooth bounded rewards through exponential shaping. Define

(21) $$d_{\min}^{\mathrm{fa}}\left(S_{t}\right)=\min_{\begin{array}{c} i\in N^{f},\;\sigma_{i}^{t}=1\;j\in N^{a},\;\sigma_{j}^{t}=1 \end{array}}∥p_{i}^{t}-p_{j}^{t}{∥}_{2},$$

which is the minimum separation between active pursuers and evaders at step *t*, and $\mathrm{dist}\left(p,\Omega_{\mathrm{obs}}\right)\;=\;\inf_{q\in \Omega_{\mathrm{obs}}}∥p-q{∥}_{2}.$ Using $\mathrm{dist}(\cdot ,\Omega_{\mathrm{obs}})$, we define a conservative safety clearance that accounts for both obstacle proximity and intra-team separation. This design penalizes near-collision configurations before an actual violation occurs. The pursuer-team safety margin with respect to obstacles and intra-team separation is

(22) $$d_{\min}^{\mathrm{safe}}\left(S_{t}\right)=\min\left\{\min_{\begin{array}{c} k\in N^{f}\;\sigma_{k}^{t}=1 \end{array}}\mathrm{dist}\left(p_{k}^{t},\Omega_{\mathrm{obs}}\right),\min_{\begin{array}{c} i,\ell \in N^{f}\;i\neq \ell ,\;\sigma_{i}^{t}=\sigma_{\ell }^{t}=1 \end{array}}∥p_{i}^{t}-p_{\ell }^{t}{∥}_{2}\right\}.$$

With desired safety distance $\delta_{\mathrm{safe}}>0$, define

(23) $$\begin{array}{cc} r_{\mathrm{cap}}\left(S_{t}\right) & =\exp\;\left(-\alpha_{\mathrm{cap}}\;d_{\min}^{\mathrm{fa}}\left(S_{t}\right)\right), \end{array}$$

(24) $$\begin{array}{cc} r_{\mathrm{surv}}\left(S_{t}\right) & =\frac{1}{N_{f}}\sum_{k\in N^{f}}\sigma_{k}^{t}\;=\;\frac{N_{\mathrm{surv}}^{t}}{N_{f}}, \end{array}$$

(25) $$\begin{array}{cc} r_{\mathrm{safe}}\left(S_{t}\right) & =\exp\;\left(-\alpha_{\mathrm{safe}}\;\max\left\{0,\;\delta_{\mathrm{safe}}-d_{\min}^{\mathrm{safe}}\left(S_{t}\right)\right\}\right), \end{array}$$

(26) $$\begin{array}{cc} r_{\mathrm{ctrl}}\left(u_{t}^{f}\right) & =\exp\;\left(-\alpha_{\mathrm{ctrl}}\;\frac{1}{N_{f}}\sum_{i\in N^{f}}∥a_{i}^{t}{∥}_{2}^{2}\right), \end{array}$$

where $r_{\mathrm{cap}}$ provides a dense signal toward engagement, $r_{\mathrm{surv}}$ rewards maintaining active pursuers, $r_{\mathrm{safe}}$ penalizes clearance shrinkage below $\delta_{\mathrm{safe}}$, and $r_{\mathrm{ctrl}}$ regularizes control effort for smoother and more robust behavior. The pursuer-team joint action is denoted by $u_{t}^{f}:={\left\{u_{i}^{t}\right\}}_{i\in N^{f}}$. These terms are aggregated as

(27) $$\begin{array}{cc} r\left(S_{t},u_{t}^{f},u_{t}^{a}\right) & =w_{\mathrm{cap}}\;r_{\mathrm{cap}}\left(S_{t}\right)+w_{\mathrm{surv}}\;r_{\mathrm{surv}}\left(S_{t}\right)+w_{\mathrm{safe}}\;r_{\mathrm{safe}}\left(S_{t}\right)+w_{\mathrm{ctrl}}\;r_{\mathrm{ctrl}}\left(u_{t}^{f}\right). \end{array}$$

The weights $\left\{w_{\mathrm{cap}},w_{\mathrm{surv}},w_{\mathrm{safe}},w_{\mathrm{ctrl}}\right\}$ control the trade-off between capture efficiency and safety, while keeping all reward components bounded and comparable in scale.

We next define the terminal-event indicators. To avoid double counting when the dwell condition persists over several steps, we count only newly occurred capture and loss events. Specifically, an event is registered only when the corresponding survival indicator changes between two consecutive steps. Let

(28) $$K_{\mathrm{cap}}^{t}=\sum_{j\in N^{a}}I\left(\sigma_{j}^{t-1}=1,\;\sigma_{j}^{t}=0\right),$$

and

(29) $$K_{\mathrm{loss}}^{t}\;=\;\sum_{i\in N^{f}}I\left(\sigma_{i}^{t-1}=1,\;\sigma_{i}^{t}=0\right).$$

For initialization, we set $\sigma_{k}^{-1}:=\sigma_{k}^{0}=1$ so that no capture or loss is counted at t=0. Then

(30) $$N_{\mathrm{cap}}^{T}\;=\;\sum_{t=0}^{T}K_{\mathrm{cap}}^{t},\;N_{\mathrm{loss}}^{T}\;=\;\sum_{t=0}^{T}K_{\mathrm{loss}}^{t}\;=\;N_{f}-N_{\mathrm{surv}}^{T}.$$

Successful disengagement at terminal time *T* is defined by

(31) $$I_{\mathrm{esc}}^{T}\;=\;\left\{\begin{array}{cc} 1, & \mathrm{if}\;N_{\mathrm{surv}}^{T}\geq 1,\;\exists \;j\in N^{a}\;s.t.\;\sigma_{j}^{T}=1,{∥p_{j}^{T}-p_{i}^{T}∥}_{2}>R_{\mathrm{eng}},\;\forall \;i\in N^{f}\;s.t.\;\sigma_{i}^{T}=1,\\ 0, & \mathrm{otherwise}. \end{array}\right.$$

An episode terminates when all evaders are captured, all pursuers are lost, or the time index reaches $T_{\max}$. The terminal reward is then given by

(32) $$r_{T}\left(S_{T}\right)\;=\;R_{\mathrm{cap}}\;N_{\mathrm{cap}}^{T}\;+\;R_{\mathrm{esc}}\;I_{\mathrm{esc}}^{T}\;-\;R_{\mathrm{loss}}\;N_{\mathrm{loss}}^{T}.$$

The evader team is treated as part of the environment and follows a non-stationary policy drawn from $\Pi^{a}$. Each pursuer agent $i\in N^{f}$ learns a decentralized policy $\pi_{i}$ conditioned on its observation history $h_{i}^{t}$. The resulting policy set is $\pi ={\left\{\pi_{i}\right\}}_{i\in N^{f}}$. It is trained to maximize the return accumulated over nonterminal steps:

(33) $$J\left(\pi \right)\;=\;E\left[\sum_{t=0}^{T-1}\gamma^{t}\;r\left(S_{t},u_{t}^{f},u_{t}^{a}\right)\;+\;\gamma^{T}\;r_{T}\left(S_{T}\right)\right],$$

where the terminal reward is applied only once at termination to avoid ambiguous terminal-step accounting.

## 4. Method

**Figure 2:**
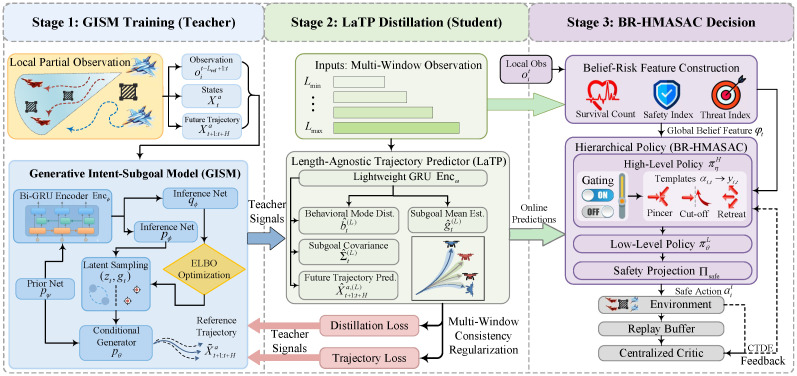
Overview. Stage 1 trains GISM by maximizing the ELBO over $\left(z_{t},g_{t}\right)$ and short-horizon trajectories. Stage 2 trains LaTP to output $\left({\hat{b}}_{t}^{\left(L\right)},{\hat{g}}_{t}^{\left(L\right)},{\hat{\Sigma }}_{t}^{\left(L\right)}\right)$ and ${\hat{X}}_{t+1:t+H}^{a,(L)}$ consistently across observation-window lengths. Stage 3 constructs belief–risk features $\varphi_{t}$ for hierarchical template mixing and applies the safety projection $\Pi_{\mathrm{safe}}$ within BR-HMASAC under CTDE.

A belief–risk-gated hierarchical multi-agent policy optimization framework is developed for cooperative multi-UAV pursuit–evasion under partial observability and non-stationary interactions. Section 4.1 develops opponent-intent inference by learning the evader’s behavior mode and subgoal from short observation windows $o_{i}^{t-L+1:t}$ with an observation-driven latent-variable generative model. Section 4.2 distills these latent teacher signals into a lightweight online predictor that operates across arbitrary observation-window lengths through multi-window distillation and cross-length consistency regularization. Section 4.3 develops safe cooperative decision making by constructing belief–risk features from the predictor outputs and training a two-level policy with maneuver-template gating, continuous control, and safety projection under centralized training with decentralized execution. Figure 2 summarizes the overall pipeline.

### 4.1. Opponent-Intent Inference via Observation-Driven Behavior-Mode and Subgoal Modeling

**Figure 3:**
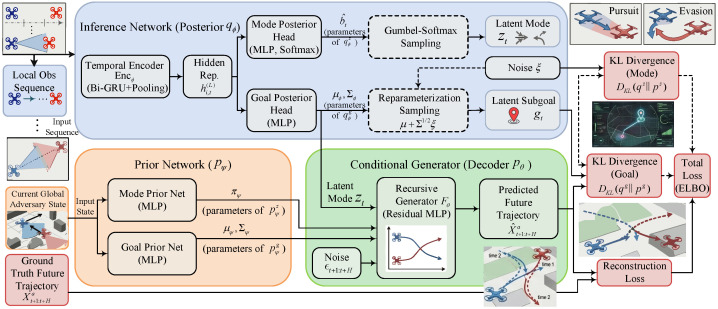
GISM. A temporal encoder maps $o_{i}^{t-L+1:t}$ to $h_{i,t}^{\left(L\right)}$. The inference network $q_{\phi }$ yields posteriors over $z_{t}$ and $g_{t}$ through Gumbel–Softmax and reparameterization, while the prior $p_{\psi }$ provides situation-dependent priors. The conditional generator $p_{\theta }$ produces $X_{t+1:t+H}^{a}$ conditioned on $\left(z_{t},g_{t}\right)$ and is trained by ELBO maximization.

An observation-driven latent-variable generative model, termed the Generative Intent–Subgoal Model (GISM), is constructed to infer the evader’s behavior mode and subgoal as $\left(z_{t},g_{t}\right)$ from short observation windows and to model the short-horizon future-trajectory distribution conditioned on $\left(z_{t},g_{t}\right)$, as shown in Figure 3.

#### 4.1.1. Local Observation Encoding and Latent-Variable Conditional Generation

Given a short observation-window sequence $o_{i}^{t-L+1:t}=\left(o_{i}^{t-L+1},\ldots ,o_{i}^{t}\right),$ a temporal representation is computed as

(34) $$h_{i,t}^{\left(L\right)}={\mathrm{Enc}}_{\phi }\left(o_{i}^{t-L+1:t}\right)\in R^{d_{h}},$$

where ${\mathrm{Enc}}_{\phi }$ is a temporal encoder, implemented as a Bi-GRU with pooling in our experiments. This mapping converts time-indexed local observations into a fixed-dimensional summary that supports amortized inference under intermittent observability and variable dynamics.

Let $N^{a}=\left\{1,\ldots ,N_{a}\right\}$ and define the evader joint state

(35) $$X_{t}^{a}={\left\{x_{j}^{t}\right\}}_{j\in N^{a}},\;x_{j}^{t}=\left[\begin{array}{c} p_{j}^{t}\\ v_{j}^{t} \end{array}\right],$$

and the future sequence over prediction horizon *H* as $X_{t+1:t+H}^{a}=\left(X_{t+1}^{a},\ldots ,X_{t+H}^{a}\right)$. Here, $X_{t}^{a}$ and $X_{t+1:t+H}^{a}$ are used for offline supervision during self-play data collection, whereas online inference relies only on $o_{i}^{t-L+1:t}$. A discrete behavior mode $z_{t}\in Ƶ=\left\{1,\ldots ,K_{z}\right\}$ and a continuous subgoal $g_{t}\in G\subset R^{d_{g}}$ are introduced. Under $N_{a}=2$,

(36) $$g_{t}=\left[\begin{array}{c} g_{t,1}\\ g_{t,2} \end{array}\right]\in R^{4},\;g_{t,j}\in R^{2}.$$

Here, $g_{t,j}$ is interpreted as a compact subgoal for evader *j* over the short horizon, so that different future modes can be represented by distinct subgoal hypotheses even when the short observation window is ambiguous.

A conditional generative process is modeled as

(37) $$\begin{array}{cc} p_{\theta ,\psi }\;\left(z_{t},g_{t},X_{t+1:t+H}^{a}\;|\;X_{t}^{a}\right) & =p_{\psi }\;\left(z_{t},g_{t}\;|\;X_{t}^{a}\right)p_{\theta }\;\left(X_{t+1:t+H}^{a}\;|\;X_{t}^{a},z_{t},g_{t}\right), \end{array}$$

which separates a situation-dependent latent prior from a latent-conditioned short-horizon generator. The situation-dependent prior factorizes as

(38) $$p_{\psi }\left(z_{t},g_{t}\;|\;X_{t}^{a}\right)=p_{\psi }^{z}\left(z_{t}\;|\;X_{t}^{a}\right)\;p_{\psi }^{g}\left(g_{t}\;|\;z_{t},X_{t}^{a}\right),$$

with

(39) $$p_{\psi }^{z}\left(z_{t}|X_{t}^{a}\right)=\mathrm{Cat}\left(\pi_{\psi }\left(X_{t}^{a}\right)\right),\;\pi_{\psi }\left(X_{t}^{a}\right)\in \Delta \left(Ƶ\right),$$

(40) $$p_{\psi }^{g}\left(g_{t}|z_{t},X_{t}^{a}\right)=N\left(\mu_{\psi }\left(z_{t},X_{t}^{a}\right),\;\Sigma_{\psi }\left(z_{t},X_{t}^{a}\right)\right).$$

This design allows the prior to adapt to the instantaneous evader state while enabling each behavior mode to carry a distinct subgoal distribution, thereby promoting interpretable multimodality.

The conditional generator is applied recursively with noise $\epsilon_{t+1:t+H}$:

(41) $$\begin{array}{cc} X_{t+\tau }^{a} & =F_{\theta }\left(X_{t+\tau -1}^{a},z_{t},g_{t},\epsilon_{t+\tau }\right),\;\tau =1,\ldots ,H, \end{array}$$

where $F_{\theta }$ is an MLP and $\epsilon_{t+\tau }$ captures stochasticity. In this way, $g_{t}$ persistently shapes the generated horizon segment toward a mode-dependent subgoal. Notably, the same $\left(z_{t},g_{t}\right)$ is shared across the *H*-step rollout, encoding the assumption that the evader’s behavior mode and subgoal remain locally consistent over the short horizon.

#### 4.1.2. Observation-Driven Inference and Variational Learning Objective

For online use under partial observability, an observation-driven approximate posterior $q_{\phi }\left(z_{t},g_{t}|o_{i}^{t-L+1:t}\right)$ is adopted, with factorization

(42) $$q_{\phi }\left(z_{t},g_{t}\;|\;o_{i}^{t-L+1:t}\right)=q_{\phi }^{z}\left(z_{t}\;|\;o_{i}^{t-L+1:t}\right)\;q_{\phi }^{g}\left(g_{t}\;|\;z_{t},o_{i}^{t-L+1:t}\right),$$

so that behavior-mode inference provides a discrete hypothesis that conditions a continuous subgoal posterior, where

(43) $$\begin{array}{cc} q_{\phi }^{z}\left(z_{t}\;|\;o_{i}^{t-L+1:t}\right) & =\mathrm{Cat}\left({\hat{b}}_{t}\right),{\hat{b}}_{t}=\mathrm{softmax}\;\left(W_{z}h_{i,t}^{\left(L\right)}+b_{z}\right), \end{array}$$

(44) $$\begin{array}{cc} q_{\phi }^{g}\left(g_{t}\;|\;z_{t},o_{i}^{t-L+1:t}\right) & =N\left(\mu_{\phi }\left(z_{t},o_{i}^{t-L+1:t}\right),\;\Sigma_{\phi }\left(z_{t},o_{i}^{t-L+1:t}\right)\right). \end{array}$$

The following quantities are used as point estimates and uncertainty measures for subsequent belief–risk construction:

(45) $$\begin{array}{c} {\hat{z}}_{t}=\arg\max_{z\in Ƶ}{\hat{b}}_{t}\left(z\right),{\hat{g}}_{t}=\mu_{\phi }\left({\hat{z}}_{t},o_{i}^{t-L+1:t}\right),{\hat{\Sigma }}_{t}=\Sigma_{\phi }\left({\hat{z}}_{t},o_{i}^{t-L+1:t}\right). \end{array}$$

In particular, ${\hat{\Sigma }}_{t}$ quantifies the dispersion of the inferred subgoal hypothesis and will later be used to conservatively inflate contact margins.

For a training tuple $\left(X_{t}^{a},o_{i}^{t-L+1:t},X_{t+1:t+H}^{a}\right)$, the evidence lower bound (ELBO) is maximized:

(46) $$\begin{array}{cc} \; & \log p_{\theta ,\psi }\;\left(X_{t+1:t+H}^{a}|X_{t}^{a}\right)\\ \; & \;\geq \;E_{q_{\phi }}\left[\log p_{\theta }\;\left(X_{t+1:t+H}^{a}|X_{t}^{a},z_{t},g_{t}\right)\right]-\;D_{\mathrm{KL}}\;\left(q_{\phi }\;\left(z_{t},g_{t}|o_{i}^{t-L+1:t}\right)\;∥\;p_{\psi }\;\left(z_{t},g_{t}|X_{t}^{a}\right)\right)\\ \; & \;≜\;L_{\mathrm{ELBO}}\left(\theta ,\phi ,\psi \right). \end{array}$$

This objective couples an observation-driven posterior with a state-conditioned prior so that inference remains feasible online while the intermediate representations remain grounded in evader dynamics observed during self-play.

Using (42), the Kullback–Leibler (KL) term decomposes as

(47) $$\begin{array}{cc} \; & D_{\mathrm{KL}}\left(q_{\phi }\left(z_{t},g_{t}|o_{i}^{t-L+1:t}\right)\;∥\;p_{\psi }\left(z_{t},g_{t}|X_{t}^{a}\right)\right)\\ \; & =D_{\mathrm{KL}}\left(q_{\phi }^{z}\left(z_{t}|o_{i}^{t-L+1:t}\right)\;∥\;p_{\psi }^{z}\left(z_{t}|X_{t}^{a}\right)\right)+E_{q_{\phi }^{z}}\left[D_{\mathrm{KL}}\left(q_{\phi }^{g}\left(g_{t}|z_{t},o_{i}^{t-L+1:t}\right)\;∥\;p_{\psi }^{g}\left(g_{t}|z_{t},X_{t}^{a}\right)\right)\right]. \end{array}$$

The first KL term regularizes the inferred mode belief toward the situation-dependent prior, while the second aligns the mode-conditional subgoal posterior with the corresponding prior component.

Reparameterization is applied to $g_{t}$:

(48) $$g_{t}=\mu_{\phi }\left(z_{t},o_{i}^{t-L+1:t}\right)+\Sigma_{\phi }^{1/2}\left(z_{t},o_{i}^{t-L+1:t}\right)\;\xi ,\xi ∼N\left(0,I\right),$$

where $\Sigma_{\phi }^{1/2}$ can be implemented through a Cholesky factor to ensure numerical stability, and Gumbel–Softmax is used for differentiable sampling of $z_{t}$.

#### 4.1.3. Offline Training and Online Inference Interface

A self-play dataset is constructed as

(49) $$D_{\mathrm{gen}}={\left\{\left(X_{t}^{a,(n)},\;o_{i}^{t-L+1:t,(n)},\;X_{t+1:t+H}^{a,(n)}\right)\right\}}_{n=1}^{N_{\mathrm{gen}}},$$

where each sample pairs a local observation window with the corresponding evader state-transition segment. To improve reproducibility, we further specify the generation protocol of the dataset $D_{\mathrm{gen}}$. Rather than being collected from an external source, this dataset is automatically constructed from self-play rollouts in the same pursuit–evasion simulator used in Section 5. Specifically, for each valid time step *t* in each self-play episode, a local observation window of length *L*, denoted by $o_{i,t-L+1:t}$, is extracted from the pursuer’s observation history and paired with the evader joint state $X_{t}^{a}$ at the same time step and the subsequent *H*-step ground-truth evader trajectory segment $X_{t+1:t+H}^{a}$ to form a supervised sample. During LaTP training, the observation-window length is selected from the representative set $L_{t}=\left\{L_{\min},L_{\mathrm{mid}},L_{\max}\right\}$ so as to explicitly cover short-, medium-, and long-window input conditions. In this way, $D_{\mathrm{gen}}$ provides offline supervision for short-window intent–subgoal generation in GISM, while also supplying multi-window distillation samples for LaTP. During data collection, obstacle layouts, initial relative configurations, and observation-window lengths are randomized to cover representative cases involving intermittent visibility, fluctuating window lengths, and behavior-mode switching. The resulting dataset contains $N_{\mathrm{epi}}=4000$ self-play episodes and approximately $N_{\mathrm{gen}}=1.0\times 10^{6}$ samples, which are split at the episode level into training, validation, and test subsets with a ratio of 8:1:1 to avoid information leakage caused by temporally adjacent windows appearing in different subsets.

The summed ELBO is maximized:

(50) $$\max_{\theta ,\phi ,\psi }\;\;\sum_{n=1}^{N_{\mathrm{gen}}}L_{\mathrm{ELBO}}^{\left(n\right)}\left(\theta ,\phi ,\psi \right).$$

During training, a fixed reference observation-window length $L_{\mathrm{ref}}$ is used to standardize the teacher-inference pathway and avoid entangling window-length effects with the generative model itself. The variational training procedure of GISM is summarized in Algorithm 1.
**Algorithm 1** Variational training of the observation-driven evader intent–subgoal generative model (GISM)  1:**Input:** dataset $D_{\mathrm{gen}}$; batch size *B*; learning rate η; reference window length $L_{\mathrm{ref}}$; horizon *H*  2:**Output:** trained parameters θ,ψ,ϕ  3:Initialize θ,ψ,ϕ; initialize Adam optimizer for (θ,ψ,ϕ) with lr η  4:Set Gumbel–Softmax temperature $\tau_{g}$ and gradient clipping threshold $g_{\max}$ (if used)  5:**while** not converged **do**  6:   Sample a mini-batch ${\left\{\left(X_{t}^{a},o_{i}^{t-L_{\mathrm{ref}}+1:t},X_{t+1:t+H}^{a}\right)\right\}}_{b=1}^{B}$ from $D_{\mathrm{gen}}$  7:   **Posterior inference:** compute $h_{i,t}^{\left(L_{\mathrm{ref}}\right)}={\mathrm{Enc}}_{\phi }\left(o_{i}^{t-L_{\mathrm{ref}}+1:t}\right)$  8:   Compute mode posterior $q_{\phi }^{z}\left(z_{t}|o_{i}^{t-L_{\mathrm{ref}}+1:t}\right)=\mathrm{Cat}\left({\hat{b}}_{t}\right)$ by (43)  9:   Sample ${\tilde{z}}_{t}$ via Gumbel–Softmax with temperature $\tau_{g}$ (straight-through optional)10:   Compute subgoal posterior $q_{\phi }^{g}\left(g_{t}|{\tilde{z}}_{t},o_{i}^{t-L_{\mathrm{ref}}+1:t}\right)=N\left(\mu_{\phi },\Sigma_{\phi }\right)$ by (44)11:   Sample $g_{t}=\mu_{\phi }+\Sigma_{\phi }^{1/2}\xi ,\;\xi ∼N\left(0,I\right)$12:   **Prior:** compute $p_{\psi }^{z}\left(z_{t}|X_{t}^{a}\right)$ and $p_{\psi }^{g}\left(g_{t}|z_{t},X_{t}^{a}\right)$ by (39) and (40)13:   **Conditional generation:** roll out $p_{\theta }\left(X_{t+1:t+H}^{a}|X_{t}^{a},{\tilde{z}}_{t},g_{t}\right)$ using $F_{\theta }$ for τ=1,…,H to obtain ${\tilde{X}}_{t+1:t+H}^{a}$14:   **ELBO terms:** compute reconstruction term $\log p_{\theta }\left(X_{t+1:t+H}^{a}|X_{t}^{a},{\tilde{z}}_{t},g_{t}\right)$ and KL term by (46) (decomposition (47) optional)15:   Form minibatch objective $L_{\mathrm{ELBO}}\left(\theta ,\phi ,\psi \right)$ and loss $L\leftarrow -L_{\mathrm{ELBO}}$16:   Backpropagate $\nabla_{\theta ,\phi ,\psi }L$; apply gradient clipping to $g_{\max}$ if used17:   Update (θ,ψ,ϕ) by one Adam step with lr η18:   Optionally anneal $\tau_{g}$ (e.g., $\tau_{g}\leftarrow \max\left(\tau_{\min},\lambda \tau_{g}\right)$)19:**end while**20:**return**θ,ψ,ϕ

### 4.2. Length-Agnostic Online Prediction via Multi-Window Distillation and Cross-Length Consistency Regularization

**Figure 4:**
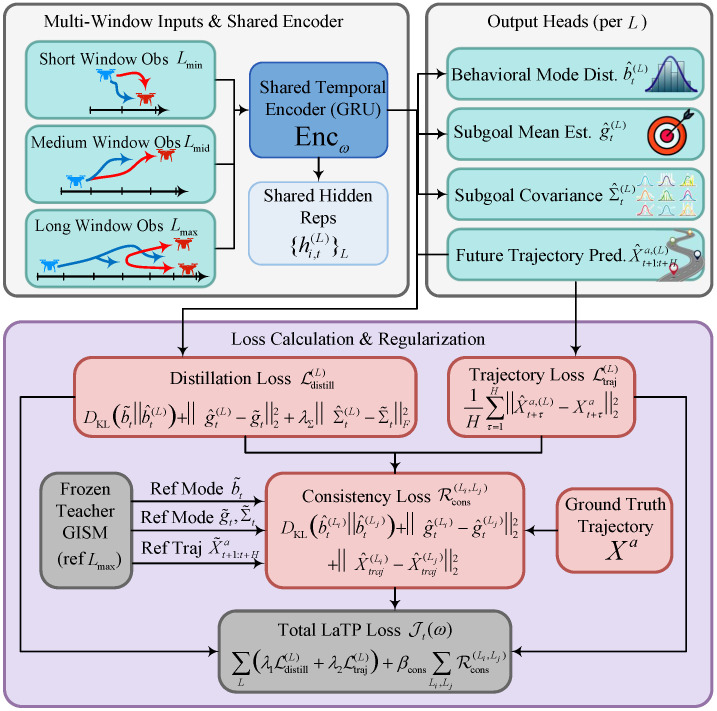
LaTP. A shared encoder ${\mathrm{Enc}}_{\omega }$ processes multi-length observation windows, and four heads output $\left({\hat{b}}_{t}^{\left(L\right)},{\hat{g}}_{t}^{\left(L\right)},{\hat{\Sigma }}_{t}^{\left(L\right)},{\hat{X}}_{t+1:t+H}^{a,(L)}\right)$. A frozen GISM provides soft teacher signals $\left({\tilde{b}}_{t},{\tilde{g}}_{t},{\tilde{\Sigma }}_{t},{\tilde{X}}_{t+1:t+H}^{a}\right)$ computed at $L_{\max}$, together with trajectory regression and cross-length consistency regularization.

GISM infers the evader’s behavior mode and subgoal $\left(z_{t},g_{t}\right)$ from a fixed reference window $L_{\mathrm{ref}}$, which may lead to unstable estimates when the observation-window length shrinks abruptly and incurs non-negligible online computational cost because of the full prior–posterior–generator forward pass. In addition, the pursuers require high-frequency updates of these intermediate representations to support timely policy switching under intermittent observability. GISM is therefore distilled into a lightweight length-agnostic predictor $F_{\omega }$, termed LaTP, which directly outputs the mode belief, subgoal with uncertainty, and an *H*-step future trajectory prediction from any available observation-window length $L\in [L_{\min},L_{\max}]$, as shown in Figure 4. This student network preserves the teacher signals while removing the fixed-window constraint at inference time. LaTP introduces no additional modeling assumption. Instead, it transfers the inference capability of GISM into a high-frequency online front end for subsequent belief–risk construction and CTDE policy learning in Section 4.3.

#### 4.2.1. Network Architecture and Input–Output Definitions

LaTP consists of an encoder ${\mathrm{Enc}}_{\omega }$ and four output heads. For any $L\in [L_{\min},L_{\max}]$,

(51) $$h_{i,t}^{\left(L\right)}={\mathrm{Enc}}_{\omega }\left(o_{i}^{t-L+1:t}\right)\in R^{d_{h}},$$

where ${\mathrm{Enc}}_{\omega }$ is a GRU-based temporal encoder, initialized from ${\mathrm{Enc}}_{\phi }$ and then fine-tuned. The shared encoder ensures that observation windows of different lengths are embedded into a common latent feature space, thereby supporting consistent downstream prediction heads. The concrete architecture and training settings of LaTP are summarized in Table 2. In the implementation used here, the encoder has two GRU layers with hidden size 128, and each prediction head is a single linear map. Given $h_{i,t}^{\left(L\right)}$, the four heads produce

(52) $$\begin{array}{cc} {\hat{b}}_{t}^{\left(L\right)} & =\mathrm{softmax}\;\left(W_{z}^{\omega }h_{i,t}^{\left(L\right)}+b_{z}^{\omega }\right)\in \Delta \left(Ƶ\right), \end{array}$$

(53) $$\begin{array}{cc} {\hat{g}}_{t}^{\left(L\right)} & =W_{g}^{\omega }h_{i,t}^{\left(L\right)}+b_{g}^{\omega }\in R^{d_{g}}, \end{array}$$

(54) $$\begin{array}{cc} {\hat{\Sigma }}_{t}^{\left(L\right)} & =\mathrm{Diag}\left(\exp(W_{\Sigma }^{\omega }h_{i,t}^{\left(L\right)}+b_{\Sigma }^{\omega })\right)\in R^{d_{g}\times d_{g}}, \end{array}$$

(55) $$\begin{array}{cc} {\hat{X}}_{t+1:t+H}^{a,(L)} & =\mathrm{Reshape}\left(W_{\mathrm{traj}}^{\omega }h_{i,t}^{\left(L\right)}+b_{\mathrm{traj}}^{\omega }\right), \end{array}$$

where Reshape(·) maps the vector output to an *H*-step evader-state sequence. A diagonal covariance head is adopted to guarantee positive semi-definiteness with minimal overhead, which is sufficient for conservative risk inflation in the setting considered here.

#### 4.2.2. Teacher Signals and Multi-Window Objectives

The trained GISM parameters (θ,ψ,ϕ) are frozen as a teacher. To eliminate window-length dependence in the teacher, teacher signals are always computed at the longest observation-window length $L_{\max}$, which serves as the most informative reference for the same trajectory segment:

(56) $${\tilde{b}}_{t}=q_{\phi }^{z}(z_{t}|o_{i}^{t-L_{\text{max}}+1:t}),$$

(57) $${\tilde{z}}_{t}=\arg\max_{z\in Ƶ}{\tilde{b}}_{t}(z),$$

(58) $${\tilde{g}}_{t}=\mu_{\phi }({\tilde{z}}_{t},o_{i}^{t–L_{\text{max}}+1:t}),$$

(59) $${\tilde{\Sigma }}_{t}=\Sigma_{\phi }({\tilde{z}}_{t},o_{i}^{t–L_{\text{max}}+1:t}),$$

(60) $${\tilde{X}}_{t+1:t+H}^{a}=E_{p_{\theta }(\cdot |X_{t}^{a},{\tilde{z}}_{t},{\tilde{g}}_{t})}[X_{t+1:t+H}^{a}],$$

where ${\tilde{X}}_{t+1:t+H}^{a}$ is implemented through a deterministic generator pass; for example, with zero noise, to obtain a smooth reference. These soft teacher signals preserve uncertainty-aware intermediate representations beyond hard mode labels, which is important when short observation windows induce ambiguous futures.

For each time step *t*, LaTP is trained on a small set of observation-window lengths

(61) $$L_{t}=\left\{L_{\min},\;L_{\mathrm{mid}},\;L_{\max}\right\},\;L_{\mathrm{mid}}=\left\lfloor \frac{L_{\min}+L_{\max}}{2}\right\rfloor .$$

For each $L\in L_{t}$, LaTP outputs

(62) $$\left({\hat{b}}_{t}^{\left(L\right)},{\hat{g}}_{t}^{\left(L\right)},{\hat{\Sigma }}_{t}^{\left(L\right)},{\hat{X}}_{t+1:t+H}^{a,(L)}\right)=F_{\omega }\left(o_{i}^{t-L+1:t}\right).$$

Training on a small fixed set $L_{t}$ provides explicit supervision at representative lengths while keeping the optimization cost manageable.

(a) Teacher-Alignment Losses

The mode posterior is distilled via

(63) $$L_{\mathrm{mode}}^{\left(L\right)}=D_{\mathrm{KL}}\left({\tilde{b}}_{t}\;∥\;{\hat{b}}_{t}^{\left(L\right)}\right),$$

so that LaTP matches the teacher’s belief distribution rather than only the argmax label. The subgoal statistics are regressed via

(64) $$L_{\mathrm{goal}}^{\left(L\right)}=∥{\hat{g}}_{t}^{\left(L\right)}-{\tilde{g}}_{t}{∥}_{2}^{2}+\lambda_{\Sigma }\;∥{\hat{\Sigma }}_{t}^{\left(L\right)}-{\tilde{\Sigma }}_{t}{∥}_{F}^{2},$$

which transfers both the subgoal mean and its dispersion to support downstream risk-aware decision-making. The trajectory head is fitted to the data via

(65) $$L_{\mathrm{traj}}^{\left(L\right)}=\frac{1}{H}\sum_{\tau =1}^{H}∥{\hat{X}}_{t+\tau }^{a,(L)}-X_{t+\tau }^{a}{∥}_{2}^{2}.$$

This term anchors the predictor to physically consistent rollouts, preventing degenerate solutions that match the teacher signals while drifting away from the ground-truth dynamics.

(b) Cross-Length Consistency

To make LaTP robust to observation-window shrinkage, its multi-length outputs are further regularized by enforcing consistency across different *L* sampled at the same time step. For any pair $L_{1}<L_{2}$ in $L_{t}$, the mode belief, subgoal statistics, and future trajectory predictions are encouraged to remain consistent under truncation, up to unavoidable information loss. Specifically, belief drift is first penalized through a symmetric KL divergence

(66) $$R_{\mathrm{mode}}^{\left(L_{1},L_{2}\right)}=D_{\mathrm{KL}}\left({\hat{b}}_{t}^{\left(L_{1}\right)}\;∥\;{\hat{b}}_{t}^{\left(L_{2}\right)}\right)+D_{\mathrm{KL}}\left({\hat{b}}_{t}^{\left(L_{2}\right)}\;∥\;{\hat{b}}_{t}^{\left(L_{1}\right)}\right),$$

which discourages window-induced posterior collapse or spurious mode switching. The continuous subgoal representation is then aligned by matching both the subgoal mean and its uncertainty estimate:

(67) $$R_{\mathrm{goal}}^{\left(L_{1},L_{2}\right)}=∥{\hat{g}}_{t}^{\left(L_{1}\right)}-{\hat{g}}_{t}^{\left(L_{2}\right)}{∥}_{2}^{2}+\lambda_{\Sigma }^{\mathrm{cons}}\;∥{\hat{\Sigma }}_{t}^{\left(L_{1}\right)}-{\hat{\Sigma }}_{t}^{\left(L_{2}\right)}{∥}_{F}^{2},$$

and the trajectory head is finally stabilized by matching the *H*-step predictions:

(68) $$R_{\mathrm{traj}}^{\left(L_{1},L_{2}\right)}=\frac{1}{H}\sum_{\tau =1}^{H}∥{\hat{X}}_{t+\tau }^{a,(L_{1})}-{\hat{X}}_{t+\tau }^{a,(L_{2})}{∥}_{2}^{2},$$

so that the online predictor does not change its future trajectory prediction solely because fewer observations are available. Aggregating all ordered length pairs within $L_{t}$ yields the per-time-step consistency regularizer

(69) $$R_{\mathrm{cons}}^{\left(t\right)}=\sum_{\begin{array}{c} L_{1},L_{2}\in L_{t}\;L_{1}<L_{2} \end{array}}\left(R_{\mathrm{mode}}^{\left(L_{1},L_{2}\right)}+R_{\mathrm{goal}}^{\left(L_{1},L_{2}\right)}+R_{\mathrm{traj}}^{\left(L_{1},L_{2}\right)}\right).$$

#### 4.2.3. Training Objective and Online Inference

The per-time-step objective is

(70) $$J_{t}\left(\omega \right)=\sum_{L\in L_{t}}\left(\lambda_{\mathrm{mode}}\;L_{\mathrm{mode}}^{\left(L\right)}+\lambda_{\mathrm{goal}}\;L_{\mathrm{goal}}^{\left(L\right)}+\lambda_{\mathrm{traj}}\;L_{\mathrm{traj}}^{\left(L\right)}\right)+\beta_{\mathrm{cons}}\;R_{\mathrm{cons}}^{\left(t\right)},$$

and offline training minimizes $\sum_{\left(t,n\right)}J_{t}^{\left(n\right)}\left(\omega \right)$ over the dataset using Adam. Specifically, LaTP parameters are updated by stochastic gradient descent with Adam as

(71) $$\omega \leftarrow \omega -\eta \;\mathrm{Adam}\;\left(\nabla_{\omega }\frac{1}{B}\sum_{b=1}^{B}J_{t_{b}}^{\left(n_{b}\right)}\left(\omega \right)\right),$$

where *B* is the mini-batch size and η is the learning rate. In all experiments, we use B=256 and $\eta =3\times 10^{-4}$, with global gradient clipping at 10.0. After training, online inference requires only a single forward pass of $F_{\omega }$ with the available observation-window length *L*, producing $\left({\hat{b}}_{t}^{\left(L\right)},{\hat{g}}_{t}^{\left(L\right)},{\hat{\Sigma }}_{t}^{\left(L\right)},{\hat{X}}_{t+1:t+H}^{a,(L)}\right)$ for belief–risk feature construction in Section 4.3. The multi-window distillation training procedure of LaTP is summarized in Algorithm 2.

LaTP uses the configuration in Table 2. The encoder is warm-started from the trained GISM encoder and then fine-tuned jointly with the four prediction heads. Training runs for at most 100 epochs, and the checkpoint with the lowest validation loss is retained, with early stopping if the validation loss does not improve for 10 consecutive epochs. At inference time, LaTP requires only one forward pass for the available window length *L*, which keeps the online latency low.
**Algorithm 2** Multi-window distillation training of LaTP  1:**Input:** dataset $D_{\mathrm{gen}}$; frozen teacher (θ,ψ,ϕ); $L_{t}=\left\{L_{\min},L_{\mathrm{mid}},L_{\max}\right\}$; batch size B=256; learning rate $\eta =3\times 10^{-4}$  2:    weights $\lambda_{\mathrm{mode}},\lambda_{\mathrm{goal}},\lambda_{\mathrm{traj}},\beta_{\mathrm{cons}}$; $\lambda_{\Sigma },\lambda_{\Sigma }^{\mathrm{cons}}$; maximum epochs $N_{\mathrm{ep}}=100$; patience P=10  3:**Output:** LaTP parameters ω  4:Initialize ω; warm-start ${\mathrm{Enc}}_{\omega }\leftarrow {\mathrm{Enc}}_{\phi }$; initialize Adam optimizer for ω with lr η and weight decay $10^{-5}$  5:Freeze teacher parameters (θ,ψ,ϕ)  6:**for** epoch $=1,\ldots ,N_{\mathrm{ep}}$ **do**  7:   Sample a mini-batch ${\left\{\left(X_{t}^{a},o_{i}^{t-L_{\max}+1:t},X_{t+1:t+H}^{a}\right)\right\}}_{b=1}^{B}$ from $D_{\mathrm{gen}}$  8:   **Teacher forward (at $L_{\max}$ only):** compute $\left({\tilde{b}}_{t},{\tilde{g}}_{t},{\tilde{\Sigma }}_{t},{\tilde{X}}_{t+1:t+H}^{a}\right)$ by (56)–(60)  9:   Initialize total loss J←010:   **for** each $L\in L_{t}$ **do**11:     Extract window $o_{i}^{t-L+1:t}$ and compute $h_{i,t}^{\left(L\right)}={\mathrm{Enc}}_{\omega }\left(o_{i}^{t-L+1:t}\right)$12:     Compute student outputs $\left({\hat{b}}_{t}^{\left(L\right)},{\hat{g}}_{t}^{\left(L\right)},{\hat{\Sigma }}_{t}^{\left(L\right)},{\hat{X}}_{t+1:t+H}^{a,(L)}\right)$ by (52)–(55)13:     Compute distillation losses:14:         $L_{\mathrm{mode}}^{\left(L\right)}=D_{\mathrm{KL}}\left({\tilde{b}}_{t}∥{\hat{b}}_{t}^{\left(L\right)}\right)$ by (63)15:         $L_{\mathrm{goal}}^{\left(L\right)}=∥{\hat{g}}_{t}^{\left(L\right)}-{\tilde{g}}_{t}{∥}_{2}^{2}+\lambda_{\Sigma }{∥{\hat{\Sigma }}_{t}^{\left(L\right)}-{\tilde{\Sigma }}_{t}∥}_{F}^{2}$ by (64)16:         $L_{\mathrm{traj}}^{\left(L\right)}=\frac{1}{H}\sum_{\tau =1}^{H}{∥{\hat{X}}_{t+\tau }^{a,(L)}-X_{t+\tau }^{a}∥}_{2}^{2}$ by (65)17:     Accumulate $J\leftarrow J+\lambda_{\mathrm{mode}}L_{\mathrm{mode}}^{\left(L\right)}+\lambda_{\mathrm{goal}}L_{\mathrm{goal}}^{\left(L\right)}+\lambda_{\mathrm{traj}}L_{\mathrm{traj}}^{\left(L\right)}$18:   **end for**19:   **Cross-length consistency:** compute $R_{\mathrm{cons}}^{\left(t\right)}$ using (66)–(68) with $\lambda_{\Sigma }^{\mathrm{cons}}$20:   Total objective $J\leftarrow J+\beta_{\mathrm{cons}}R_{\mathrm{cons}}^{\left(t\right)}$21:   Backpropagate $\nabla_{\omega }J$, clip the global gradient norm at 10.0, and update ω by one Adam step with lr η22:   Evaluate the validation loss and save the checkpoint if improved23:   **if** no validation improvement is observed for *P* consecutive epochs **then break**24:**end for**25:**return** ω

### 4.3. Safe Cooperative Decision Making via Belief–Risk-Gated Hierarchical Multi-Agent Reinforcement Learning

**Figure 5:**
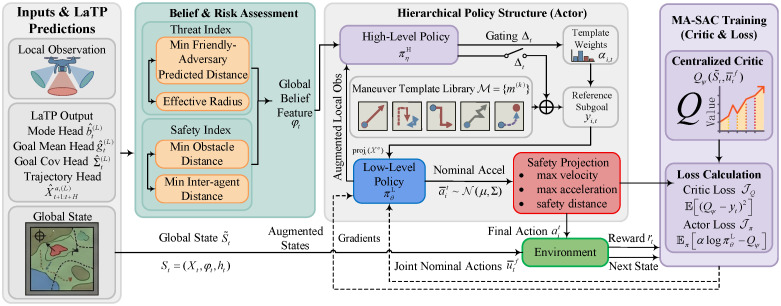
BR-HMASAC. LaTP produces belief–risk features $\varphi_{t}$; the high-level gate $\Delta_{t}$ and template weights $\alpha_{i,t}$ generate reference subgoals $y_{i,t}$; the low-level Gaussian policy outputs nominal actions ${\bar{a}}_{i}^{t}$, and $\Pi_{\mathrm{safe}}$ yields feasible controls; a centralized critic trains the hierarchy under CTDE.

Building on Section 4.1 and Section 4.2, a belief–risk-gated hierarchical multi-agent reinforcement learning module is developed that explicitly decomposes the pursuer joint policy into a high-level gating and maneuver-template policy and a low-level continuous control policy. This decision architecture is referred to as the Belief–Risk-Gated Hierarchical Multi-Agent Soft Actor-Critic (BR-HMASAC). The high-level policy leverages the evader mode belief ${\hat{b}}_{t}^{\left(L\right)}$, subgoal estimate ${\hat{g}}_{t}^{\left(L\right)}$, and future trajectory predictions ${\hat{X}}_{t+1:t+H}^{a,(L)}$ to produce a belief–risk gate and template-mixing weights, thereby generating structured reference subgoals for each pursuer. Conditioned on these references, the low-level policy outputs continuous controls and enforces dynamic and safety constraints through a safety projection layer. The entire hierarchy is optimized end-to-end using multi-agent soft actor-critic (MA-SAC) under centralized training with decentralized execution (CTDE). The belief–risk feature construction, hierarchical actor design, and CTDE training pipeline of BR-HMASAC are illustrated in Figure 5.

#### 4.3.1. Belief—Risk Features and Hierarchical Action Parameterization

(a) Contact Risk and Safety Risk

Contact risk is quantified by combining predicted evader motion with uncertainty in the inferred subgoal so that greater semantic ambiguity leads to a more conservative engagement assessment. The engagement radius is inflated using LaTP uncertainty:

(72) $$R_{\mathrm{eng}}^{\mathrm{eff}}\left(t\right)=R_{\mathrm{eng}}+\kappa_{\mathrm{eng}}\;\sqrt{\lambda_{\max}\;\left({\hat{\Sigma }}_{t}^{\left(L\right)}\right)},$$

where $\lambda_{\max}\left(\cdot \right)$ is the largest eigenvalue and $\sqrt{\lambda_{\max}\left({\hat{\Sigma }}_{t}^{\left(L\right)}\right)}$ approximates the maximum positional dispersion implied by the subgoal posterior. The horizon-wise minimum inflated contact distance is then defined as

(73) $$d_{\min}^{\mathrm{fa},\mathrm{pred}}\left(t\right)=\min_{\begin{array}{c} \tau \in \{1,\ldots ,H\}\;i\in N^{f},\;\sigma_{i}^{t}=1\;j\in N^{a},\;\sigma_{j}^{t}=1 \end{array}}\left(∥{\hat{p}}_{j}^{t+\tau ,(L)}-p_{i}^{t}{∥}_{2}-R_{\mathrm{eng}}^{\mathrm{eff}}\left(t\right)\right),$$

which evaluates the worst-case proximity between the current pursuer positions and the predicted evader positions over the horizon, yielding the contact-risk index

(74) $$J_{\mathrm{threat}}\left(t\right)=\max\left\{0,\;\delta_{\mathrm{th}}-d_{\min}^{\mathrm{fa},\mathrm{pred}}\left(t\right)\right\}.$$

For self-risk, a simple short-horizon kinematic rollout of the pursuers is used to estimate near-term clearance, thereby avoiding additional optimization inside the risk estimator:

(75) $${\bar{p}}_{k}^{t+\tau }=p_{k}^{t}+\tau \;\Delta t\;v_{k}^{t},\;\tau =1,\ldots ,H,$$

and the predicted minimum safety margin is computed as

(76) $$\begin{array}{cc} d_{\min}^{\mathrm{safe},\mathrm{pred}}\left(t\right)=\min\{ & \min_{\begin{array}{c} \tau \in \{1,\ldots ,H\}\;k\in N^{f},\;\sigma_{k}^{t}=1 \end{array}}\mathrm{dist}\left({\bar{p}}_{k}^{t+\tau },\Omega_{\mathrm{obs}}\right),\\ \; & \min_{\begin{array}{c} \tau \in \{1,\ldots ,H\}\;i,\ell \in N^{f},\;i\neq \ell \;\sigma_{i}^{t}=\sigma_{\ell }^{t}=1 \end{array}}∥{\bar{p}}_{i}^{t+\tau }-{\bar{p}}_{\ell }^{t+\tau }{∥}_{2}\}, \end{array}$$

leading to

(77) $$J_{\mathrm{safe}}\left(t\right)=\max\left\{0,\;\delta_{\mathrm{safe}}-d_{\min}^{\mathrm{safe},\mathrm{pred}}\left(t\right)\right\}.$$

(b) Belief Feature

The belief–risk feature vector is defined as

(78) $$\varphi_{t}=\left[{\hat{b}}_{t}^{\left(L\right)},\;\psi_{g}\;\left({\hat{g}}_{t}^{\left(L\right)},X_{t}\right),\;J_{\mathrm{threat}}\left(t\right),\;J_{\mathrm{safe}}\left(t\right)\right],$$

where $\psi_{g}\left(\cdot \right)$ extracts a fixed-dimensional geometric descriptor of the inferred subgoal, e.g., the relative subgoal vectors with respect to pursuer positions and their normalized distances. Per-agent augmented observations are then constructed as

(79) $${\tilde{o}}_{i}^{t}=\left[o_{i}^{t},\;\varphi_{t},\;\psi_{x}\;\left({\hat{X}}_{t+1:t+H}^{a,(L)},i\right)\right],$$

where $\psi_{x}\left(\cdot \right)$ denotes an agent-centric projection of the predicted evader trajectory.

(c) High-Level Gate and Template Mixing

The high-level module outputs a continuous gate and agent-specific template weights. This design enables smooth switching among maneuver primitives while keeping the geometric templates explicit and low-dimensional. A scalar gate is defined as

(80) $$\Delta_{t}=\sigma \;\left(w_{\Delta }^{⊤}\varphi_{t}+b_{\Delta }\right)\in \left(0,1\right),$$

and the maneuver-template set is chosen as $M=\{m^{\left(1\right)},m^{\left(2\right)},m^{\left(3\right)},m^{\left(4\right)}\}$, corresponding to pursue, encircle, lure, and disengage, respectively. In our implementation, the template bank size is fixed to K=4. Each template is implemented by a differentiable geometric generator with six scalar parameters

(81) $$\theta_{k}=\left[\omega_{k}^{\mathrm{app}},\omega_{k}^{\tan},\omega_{k}^{\mathrm{ret}},\omega_{k}^{\mathrm{sep}},\omega_{k}^{\mathrm{obs}},\nu_{k}\right],\;k=1,\ldots ,4,$$

so the full template bank contains 24 scalar parameters.

Let

(82) $$e_{i,t}^{\mathrm{app}}=\frac{{\hat{g}}_{t}^{\left(L\right)}-p_{i}^{t}}{∥{\hat{g}}_{t}^{\left(L\right)}-p_{i}^{t}{∥}_{2}+\varepsilon },\;e_{i,t}^{\tan}=s_{i}R_{\pi /2}e_{i,t}^{\mathrm{app}},\;e_{i,t}^{\mathrm{ret}}=-\;e_{i,t}^{\mathrm{app}},$$

where $R_{\pi /2}$ denotes a π/2 rotation matrix and $s_{i}\in \left\{-1,+1\right\}$ is a fixed sign used to assign opposite circling directions to the two pursuers. We further define

(83) $$e_{i,t}^{\mathrm{sep}}=\frac{p_{i}^{t}-p_{\ell^{⋆}}^{t}}{∥p_{i}^{t}-p_{\ell^{⋆}}^{t}{∥}_{2}+\varepsilon },\;\ell^{⋆}=\arg\min_{\ell \in N^{f}\setminus \left\{i\right\}}{∥p_{i}^{t}-p_{\ell }^{t}∥}_{2},$$

(84) $$e_{i,t}^{\mathrm{obs}}=\frac{p_{i}^{t}-q_{i}^{⋆}}{∥p_{i}^{t}-q_{i}^{⋆}{∥}_{2}+\varepsilon },\;q_{i}^{⋆}=\arg\min_{q\in \partial \Omega_{\mathrm{obs}}}{∥p_{i}^{t}-q∥}_{2},$$

which represent approach, tangential, retreat, teammate-separation, and obstacle-repulsion directions, respectively.

For template *k*, the direction coefficients are constrained to the simplex

(85) $$\beta^{\left(k\right)}=\mathrm{softmax}\left(\omega_{k}^{\mathrm{app}},\omega_{k}^{\tan},\omega_{k}^{\mathrm{ret}},\omega_{k}^{\mathrm{sep}},\omega_{k}^{\mathrm{obs}}\right)\in \Delta \left(\left\{1,\ldots ,5\right\}\right),$$

and the template radius is bounded by

(86) $$\rho^{\left(k\right)}=\rho_{\max}\;\sigma \left(\nu_{k}\right)\in \left(0,\rho_{\max}\right).$$

The resulting template direction is

(87) $$d_{i,t}^{\left(k\right)}=\frac{\beta_{1}^{\left(k\right)}e_{i,t}^{\mathrm{app}}+\beta_{2}^{\left(k\right)}e_{i,t}^{\tan}+\beta_{3}^{\left(k\right)}e_{i,t}^{\mathrm{ret}}+\beta_{4}^{\left(k\right)}e_{i,t}^{\mathrm{sep}}+\beta_{5}^{\left(k\right)}e_{i,t}^{\mathrm{obs}}}{{∥\beta_{1}^{\left(k\right)}e_{i,t}^{\mathrm{app}}+\beta_{2}^{\left(k\right)}e_{i,t}^{\tan}+\beta_{3}^{\left(k\right)}e_{i,t}^{\mathrm{ret}}+\beta_{4}^{\left(k\right)}e_{i,t}^{\mathrm{sep}}+\beta_{5}^{\left(k\right)}e_{i,t}^{\mathrm{obs}}∥}_{2}+\varepsilon }.$$

The geometric generator is then defined as

(88) $$y_{i,t}^{\left(k\right)}=G^{\left(k\right)}\left(X_{t},{\hat{g}}_{t}^{\left(L\right)},{\hat{X}}_{t+1:t+H}^{a,(L)}\right)=\Pi_{\Omega_{\mathrm{free}}}\left(p_{i}^{t}+\rho^{\left(k\right)}d_{i,t}^{\left(k\right)}\right)\in \Omega_{\mathrm{free}},$$

where

(89) $$\Pi_{\Omega_{\mathrm{free}}}\left(x\right):=\arg\min_{y\in \Omega_{\mathrm{free}}}{∥y-x∥}_{2}^{2}$$

projects the unconstrained reference onto the obstacle-free space. Hence, each template is differentiable with respect to its parameters, its output is kept inside $\Omega_{\mathrm{free}}$ by construction, and its step length is bounded by $\rho_{\max}$. Fine-grained dynamic and collision constraints are enforced later by the safety projection in the low-level controller.

Template weights are

(90) $$\alpha_{i,t}=\mathrm{softmax}\;\left(W_{\alpha }\;\left[{\tilde{o}}_{i}^{t};\Delta_{t}\right]+b_{\alpha }\right)\in \Delta \left(\left\{1,\ldots ,4\right\}\right),$$

and the mixed reference subgoal is

(91) $$y_{i,t}=\sum_{k=1}^{4}\alpha_{i,t}\left(k\right)\;y_{i,t}^{\left(k\right)}.$$

The high-level output is denoted by

(92) $$h_{t}=\left(\Delta_{t},\;{\left\{\alpha_{i,t}\right\}}_{i\in N^{f}}\right)=\pi_{\eta }^{H}\left(\varphi_{t},{\left\{{\tilde{o}}_{i}^{t}\right\}}_{i\in N^{f}}\right).$$

(d) Low-Level Conditional Control with Safety Projection

The low-level controller takes the augmented observation and the high-level reference as input. It first produces a nominal acceleration and then projects it onto a locally safe action set. The low-level policy samples nominal accelerations

(93) $${\bar{a}}_{i}^{t}∼\pi_{\theta }^{L}\left({\bar{a}}_{i}^{t}\;|\;{\tilde{o}}_{i}^{t},y_{i,t}\right)=N\left(\mu_{\theta }\left({\tilde{o}}_{i}^{t},y_{i,t}\right),\;\Sigma_{\theta }\left({\tilde{o}}_{i}^{t},y_{i,t}\right)\right),$$

and applies the projected action

(94) $$a_{i}^{t}=\Pi_{\mathrm{safe}}\left({\bar{a}}_{i}^{t};\;X_{t},{\hat{X}}_{t+1:t+H}^{a,(L)},\Omega_{\mathrm{obs}}\right):=\arg\min_{a\in A_{i}^{\mathrm{safe}}\left(t\right)}\frac{1}{2}{∥a-{\bar{a}}_{i}^{t}∥}_{2}^{2}.$$

The feasible set $A_{i}^{\mathrm{safe}}\left(t\right)$ enforces acceleration bounds, one-step speed bounds, obstacle clearance, and inter-pursuer separation:

(95) $$A_{i}^{\mathrm{safe}}\left(t\right)=\left\{a\in R^{2}{\;|\;∥a∥}_{2}\leq a_{i}^{\max},\;{∥v_{i}^{t}+\Delta t\;a∥}_{2}\leq v_{i}^{\max},\;h_{i,m}^{\mathrm{obs}}\left(a\right)\geq 0,\;\forall m\in M_{i}^{t},\;h_{i\ell }^{\mathrm{col}}\left(a\right)\geq 0,\;\forall \ell \in N^{f}\;\setminus \;\left\{i\right\}\right\}.$$

For projection only, we use the one-step constant-acceleration look-ahead

(96) $${\tilde{p}}_{i}^{t+1}\left(a\right)=p_{i}^{t}+\Delta t\;v_{i}^{t}+\frac{1}{2}\Delta t^{2}a,$$

which provides a control-dependent position surrogate for evaluating local safety margins. The actual environment state is still propagated by (5) after the projected action is applied.

Obstacle clearance is written as

(97) $$h_{i,m}^{\mathrm{obs}}\left(a\right)={\left(n_{i,m}^{t}\right)}^{⊤}\;\left({\tilde{p}}_{i}^{t+1}\left(a\right)-q_{i,m}^{t}\right)-\delta_{\mathrm{obs}},\;m\in M_{i}^{t},$$

where $q_{i,m}^{t}$ is the closest point on the *m*-th active obstacle boundary, $n_{i,m}^{t}$ is the corresponding outward unit normal, and $\delta_{\mathrm{obs}}>0$ is the obstacle safety margin. The active obstacle set $M_{i}^{t}$ is selected from the local maneuver corridor induced by the current reference $y_{i,t}$ and the predicted evader trajectory ${\hat{X}}_{t+1:t+H}^{a,(L)}$.

Inter-pursuer separation is enforced by

(98) $$h_{i\ell }^{\mathrm{col}}\left(a\right)={\left(n_{i\ell }^{t}\right)}^{⊤}\;\left({\tilde{p}}_{i}^{t+1}\left(a\right)-{\tilde{p}}_{\ell }^{t+1}\right)-\delta_{\mathrm{col}},\;\ell \in N^{f}\setminus \left\{i\right\},$$

where

(99) $${\tilde{p}}_{\ell }^{t+1}=p_{\ell }^{t}+\Delta t\;v_{\ell }^{t},\;n_{i\ell }^{t}=\frac{p_{i}^{t}-p_{\ell }^{t}}{∥p_{i}^{t}-p_{\ell }^{t}{∥}_{2}+\varepsilon },$$

and $\delta_{\mathrm{col}}>0$ is the minimum separation margin.

At each step, the nonlinear safety constraints are evaluated at the nominal action ${\bar{a}}_{i}^{t}$ and linearized once, which yields a two-dimensional convex quadratic program. The resulting projection is solved online with an active-set QP solver. If the linearized problem is infeasible, a backtracking fallback is used: $a_{i}^{t}=\lambda {\bar{a}}_{i}^{t}$ with the largest λ∈[0,1] that satisfies the bound constraints and the linearized safety inequalities. This makes the projection operator explicit and reproducible while keeping the online cost small.

#### 4.3.2. CTDE Learning with a Centralized Soft Critic

The hierarchy is trained with multi-agent soft actor-critic (MA-SAC) under CTDE. The implementation follows the configuration in Table 3. The high-level gate network and the template-weight network are two-layer MLPs with 128 hidden units per layer and ReLU activations. The low-level Gaussian actor is a two-layer MLP with 256 hidden units per layer. The centralized critic adopts twin Q-networks, each with two hidden layers of 256 units and ReLU activations. The actor outputs a mean vector and a diagonal log-standard deviation, with the latter clipped to [−5,2] for numerical stability. Define

$${\tilde{S}}_{t}=\left(X_{t},\varphi_{t},h_{t}\right),\;{\bar{u}}_{t}^{f}={\left\{{\bar{a}}_{i}^{t}\right\}}_{i\in N^{f}},$$

and let $Q_{\psi }\left({\tilde{S}}_{t},{\bar{u}}_{t}^{f}\right)$ be a centralized critic with target network $Q_{\bar{\psi }}$. The critic loss is

(100) $$\begin{array}{cc} J_{Q}\left(\psi \right) & =E[{\left(Q_{\psi }\left({\tilde{S}}_{t},{\bar{u}}_{t}^{f}\right)-y_{t}\right)}^{2}],\\ y_{t} & =r_{t}+\gamma \;E_{{\bar{u}}_{t+1}^{f}∼\pi_{\theta }^{L}}[Q_{\bar{\psi }}\left({\tilde{S}}_{t+1},{\bar{u}}_{t+1}^{f}\right)-\alpha \sum_{i\in N^{f}}\log\pi_{\theta }^{L}\left({\bar{a}}_{i}^{t+1}\;|\;{\tilde{o}}_{i}^{t+1},y_{i,t+1}\right)], \end{array}$$

where $\pi_{\theta }^{L}={\left\{\pi_{\theta }^{L}\left({\bar{a}}_{i}^{t}|{\tilde{o}}_{i}^{t},y_{i,t}\right)\right\}}_{i\in N^{f}}$ and α is the temperature parameter.

The low-level actor objective is

(101) $$J_{\pi }\left(\theta ,\eta \right)=E\left[\alpha \sum_{i\in N^{f}}\log\pi_{\theta }^{L}\left({\bar{a}}_{i}^{t}\;|\;{\tilde{o}}_{i}^{t},y_{i,t}\right)-Q_{\psi }\left({\tilde{S}}_{t},{\bar{u}}_{t}^{f}\right)\right],$$

and α is tuned by

(102) $$J_{\alpha }=E\left[-\alpha \left(\sum_{i\in N^{f}}\log\pi_{\theta }^{L}\left({\bar{a}}_{i}^{t}\;|\;{\tilde{o}}_{i}^{t},y_{i,t}\right)+\bar{H}\right)\right],$$

where $\bar{H}$ is the target entropy.

We optimize all trainable parameters with Adam. Specifically, critic, actor, and temperature updates are performed as

(103) $$\psi \leftarrow \psi -\eta_{Q}\;\nabla_{\psi }J_{Q}\left(\psi \right),$$

(104) $$\left(\theta ,\eta \right)\leftarrow \left(\theta ,\eta \right)-\eta_{\pi }\;\nabla_{\theta ,\eta }J_{\pi }\left(\theta ,\eta \right),$$

(105) $$\log\alpha \leftarrow \log\alpha -\eta_{\alpha }\;\nabla_{\log\alpha }J_{\alpha },$$

(106) $$\bar{\psi }\leftarrow \tau \psi +\left(1-\tau \right)\bar{\psi }.$$

In all experiments, we use $\eta_{Q}=3\times 10^{-4}$, $\eta_{\pi }=3\times 10^{-4}$, $\eta_{\alpha }=1\times 10^{-4}$, replay buffer capacity $10^{6}$, batch size 256, discount factor γ=0.99, and target-update coefficient $\tau =5\times 10^{-3}$. The entropy coefficient is tuned automatically from the initial value α=0.2 with target entropy −2 per agent. Nominal actions are sampled by the reparameterization trick during actor updates.

Gradients are backpropagated through $\left({\tilde{S}}_{t},h_{t},\left\{y_{i,t}\right\}\right)$, enabling end-to-end updates of both θ and the high-level parameters η. Training begins after a warm-up phase of 5000 environment steps. Thereafter, one gradient update is performed per environment step. Each model is trained for 1000 episodes, and the checkpoint with the highest moving-average return over the last 100 episodes is retained. The overall BR-HMASAC training procedure under CTDE is summarized in Algorithm 3.

BR-HMASAC uses the configuration in Table 3. The high-level policy and the low-level controller are trained jointly under CTDE, while LaTP is frozen during reinforcement learning. This configuration was found to provide stable critic learning, reliable entropy adaptation, and reproducible convergence across seeds.
**Algorithm 3** BR-HMASAC training under CTDE  1:**Input:** env E; LaTP $F_{\omega }$; templates ${\left\{G^{\left(k\right)}\right\}}_{k=1}^{4}$; replay buffer B  2:      discount γ=0.99; learning rates $\eta_{Q}=3\times 10^{-4}$, $\eta_{\pi }=3\times 10^{-4}$, $\eta_{\alpha }=10^{-4}$; target-mix $\tau =5\times 10^{-3}$  3:      batch size B=256; replay capacity $\left|B\right|=10^{6}$; warm-up steps $N_{\mathrm{warm}}=5000$; training episodes $N_{\mathrm{ep}}=1000$  4:**Output:**$\eta ,\theta ,\psi ,\bar{\psi }$  5:Initialize high-level policy $\pi_{\eta }^{H}$, low-level policy $\pi_{\theta }^{L}$, critic $Q_{\psi }$, target critic $Q_{\bar{\psi }}\leftarrow Q_{\psi }$, and temperature α  6:**for** episode $=1,\ldots ,N_{\mathrm{ep}}$ **do**  7:     Reset environment and initialize local histories ${\left\{h_{i,0}\right\}}_{i\in N^{f}}$  8:     **for** t=0 **to** $T_{\max}-1$ **do**  9:          **(1) Online inference:** select available window length *L* from the current history; run LaTP to obtain $\left({\hat{b}}_{t}^{\left(L\right)},{\hat{g}}_{t}^{\left(L\right)},{\hat{\Sigma }}_{t}^{\left(L\right)},{\hat{X}}_{t+1:t+H}^{a,(L)}\right)$10:          **(2) Belief–risk features:** compute $\varphi_{t}$ including $J_{\mathrm{threat}}\left(t\right)$ and $J_{\mathrm{safe}}\left(t\right)$, and construct ${\tilde{o}}_{i}^{t}$ for each *i*11:          **(3) High-level decision:** compute $\left(\Delta_{t},\alpha_{i,t}\right)=\pi_{\eta }^{H}\left(\varphi_{t},\left\{{\tilde{o}}_{i}^{t}\right\}\right)$ and generate template references $y_{i,t}^{\left(k\right)}=G^{\left(k\right)}\left(\cdot \right)$; mix $y_{i,t}=\sum_{k=1}^{4}\alpha_{i,t}\left(k\right)\;y_{i,t}^{\left(k\right)}$12:          **(4) Low-level control:** sample nominal actions ${\bar{a}}_{i}^{t}∼\pi_{\theta }^{L}\left(\cdot |{\tilde{o}}_{i}^{t},y_{i,t}\right)$ for all *i*13:          **(5) Safety projection:** apply $a_{i}^{t}=\Pi_{\mathrm{safe}}\left({\bar{a}}_{i}^{t};\;X_{t},{\hat{X}}_{t+1:t+H}^{a,(L)},\Omega_{\mathrm{obs}}\right)$ and execute the joint action ${\left\{a_{i}^{t}\right\}}_{i\in N^{f}}$14:          Step the environment and obtain reward $r_{t}$, next-state information, and termination flag done15:          Build ${\tilde{S}}_{t}=\left(X_{t},\varphi_{t},h_{t}\right)$ and ${\tilde{S}}_{t+1}$; store transition in B16:          **if** done **then**17:               **break**18:          **end if**19:          **if** the total number of environment steps $>N_{\mathrm{warm}}$ **then**20:               **(6) CTDE updates:**21:               Sample a mini-batch of size *B* from B22:               Update critic ψ by minimizing $J_{Q}\left(\psi \right)$23:               Update low-level actor θ and high-level parameters η by minimizing $J_{\pi }\left(\theta ,\eta \right)$24:               Update temperature α by minimizing $J_{\alpha }$25:               Soft update target critic $\bar{\psi }\leftarrow \tau \psi +\left(1-\tau \right)\bar{\psi }$26:        **end if**27:     **end for**28:     Evaluate the moving-average return over the last 100 episodes and save the checkpoint if improved29:**end for**30:**return** $\eta ,\theta ,\psi ,\bar{\psi }$

## 5. Experimental Results and Analysis

### 5.1. Experimental Setup and Baselines

All methods are evaluated in a 2:2 pursuit–evasion game following the system model and task formulation in Section 3. To ensure fair and reproducible comparisons, all methods share the same 2D workspace, obstacle generator, UAV dynamics, sensing and communication limits, as well as reward and termination settings. The evader side is fixed to the same policy instance $\pi_{\mathrm{fix}}^{a}\in \Pi^{a}$ during both training and evaluation so that performance differences can be attributed to pursuer-side learning rather than differences in evader implementation.

#### 5.1.1. Task Environment and Parameter Settings

Table 4 summarizes the environment and platform parameters shared by all methods, including workspace size, dynamic limits, obstacle configuration, sensing range, communication radius, and safety distances. These parameters jointly define the task difficulty, information conditions, and physical constraints, thereby supporting fair comparison and reproducibility. Table 5, by contrast, reports the algorithm-level parameters used in trajectory prediction, reward construction, and risk-index design, so as to make explicit how forecasting, efficiency–safety trade-off regulation, and constraint handling are instantiated in the proposed framework. All experiments were conducted on Windows 11 with an Intel Core i9-14900K CPU, two RTX 4090 GPUs (24 GB each), and 128 GB RAM. The simulation environment was implemented as a custom 2D pursuit–evasion simulator, with geometry-based line-of-sight checking, occlusion-aware field-of-view computation, randomized obstacle generation, and stepwise multi-agent interaction. The learning and inference modules were implemented in PyTorch 2.5, while environment stepping, reward evaluation, and safety-related geometric checks were executed in Python 3.9.

#### 5.1.2. Evader Policy Family and Experimental Protocol Under a Fixed Evader Policy

The evaders are implemented as a mode-driven motion-primitive family with Ƶ={0,1,2,3}. For each evader $j\in N^{a}$, $u_{j}^{t}∼\pi^{a}\left(\cdot |X_{t},z_{t}\right)$, where $\pi^{a}\in \Pi^{a}$. The primitive mean is clipped to satisfy acceleration bounds, and stochasticity is used only to model execution perturbations. The mode evolves according to a shared Markov kernel, $z_{t+1}∼P_{z}\left(\cdot |z_{t},X_{t}\right)$, with identical parameters across all experiments. To ensure consistent evaluation, a fixed evader policy is adopted, meaning that the same instance $\pi_{\mathrm{fix}}^{a}\in \Pi^{a}$ is used throughout both training and evaluation. For each seed s∈{0,…,9}, the same seed is reused to generate obstacle layouts, initial states, and evader perturbations for all methods. This ensures comparable episode difficulty across all experiments.

#### 5.1.3. Baseline Algorithms

The following representative multi-UAV pursuit–evasion methods are compared:

- CBC-TP Net [3]: LSTM-based target prediction combined with MADDPG-style coordination.
- OPEN [6]: an RL policy embedded in receding-horizon online planning under partial observability.
- RGATD3 [10]: spatio-temporal relational graph attention with TD3 for continuous-action pursuit.
- NAGC [11]: normalizing-flow policies with graph-attention critics for multimodal interactions.
- MSMAR-RL [27]: multi-step reach–avoid safety values with masked attention for high-speed games.

### 5.2. Overall Performance Evaluation

Overall performance is evaluated under $N_{\mathrm{obs}}\in \left\{5,8,12,15\right\}$ using expected-return convergence, reward-component decomposition, and terminal-event statistics. The evaluation metrics are chosen to match the objectives of this study at different levels. Expected return provides an overall measure of optimization effectiveness and training stability in obstacle-rich pursuit–evasion tasks. Reward-component decomposition is further used to reveal whether the performance gains arise from improvements in capture reward, survivability, safety preservation, or control smoothness, thereby clarifying the contribution of each design component. Terminal-event statistics directly reflect task-level outcomes, including capture effectiveness, pursuer survival, and suppression of evader escape. In addition, the constraint-violation rate, convergence behavior, and inference latency reported in the subsequent studies are used to assess safety, learning efficiency, and online real-time feasibility, respectively. Taken together, these metrics provide a comprehensive assessment of whether the proposed framework can preserve cooperative effectiveness under incomplete information, regulate the efficiency–safety trade-off in a controlled manner, and satisfy the timing requirements of online deployment.

Unless otherwise stated, all methods in this subsection are trained with 10 independent random seeds under the same fixed evader instance $\pi_{\mathrm{fix}}^{a}$. To avoid drawing conclusions from mean curves alone, we additionally conduct formal significance analysis on the key outcome metrics. For return convergence, we use the mean normalized J(π) over the last 100 training iterations of each seed as the post-convergence return statistic. For terminal events, we use the per-seed averages of $E[N_{\mathrm{cap}}^{T}]$, $E[N_{\mathrm{loss}}^{T}]$, and $P(I_{\mathrm{esc}}^{T}=1)$ computed from the evaluation episodes. In each obstacle setting, the proposed method is compared with the strongest baseline using Welch’s two-sample *t*-test, with Holm–Bonferroni correction for multiple comparisons at α=0.05. We also report Cohen’s *d* as the effect size.

#### 5.2.1. Convergence of Expected Return

**Figure 6:**
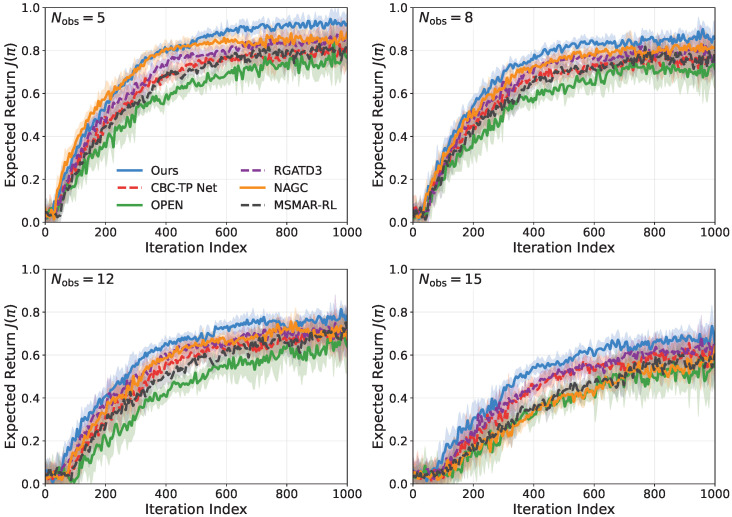
Normalized expected return J(π) under $N_{\mathrm{obs}}=5,8,12,15$ (mean ± std over 10 seeds).

Figure 6 shows the training curves over 1000 iterations with 10 seeds under $\pi_{\mathrm{fix}}^{a}$. The proposed method achieves the highest final return and the fastest convergence across all settings. For $N_{\mathrm{obs}}=5,8,12,15$, its normalized J(π) stabilizes at approximately 0.93,0.86,0.78,0.70, outperforming the strongest baseline by about 5∼7% while exhibiting a narrower variance band. Using the last-100-iteration mean return as the comparison statistic, the advantage remains significant after correction, with corrected *p*-values of 0.031,0.024,0.018,0.012 and corresponding Cohen’s *d* values of 0.95,1.08,1.21,1.35 for $N_{\mathrm{obs}}=5,8,12,15$, respectively. These results indicate moderate significance in sparse scenes and stronger separation in dense scenes, which is consistent with the increasing benefit of stable inference and risk-aware hierarchical control under more difficult conditions. These gains mainly stem from more stable inference under varying observation-window lengths and from risk-aware hierarchical switching with a safety projection layer, which reduces oscillatory exploration while preserving capture opportunities.

#### 5.2.2. Reward-Component Decomposition

**Figure 7:**
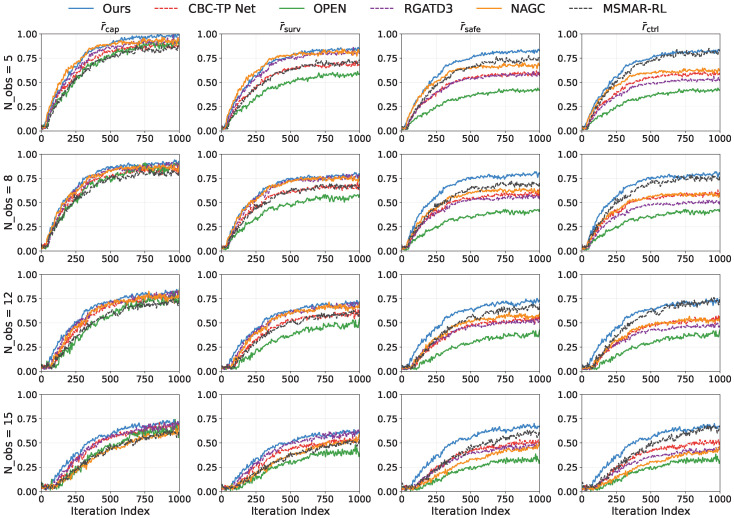
Normalized stepwise reward components (mean over 10 seeds). Rows: $N_{\mathrm{obs}}=5,8,12,15$. Columns: ${\bar{r}}_{\mathrm{cap}}$, ${\bar{r}}_{\mathrm{surv}}$, ${\bar{r}}_{\mathrm{safe}}$, ${\bar{r}}_{\mathrm{ctrl}}$.

Figure 7 decomposes the stepwise reward using the weights in Table 5. The proposed method consistently improves the capture and survival components across obstacle densities and yields the most stable safety component, indicating effective pursuit under explicit safety handling. The control-smoothness component remains comparable in sparse settings and decreases slightly in dense settings, reflecting a modest sacrifice in smoothness to improve capture and survivability under constraints.

#### 5.2.3. Terminal Event Statistics

**Figure 8:**
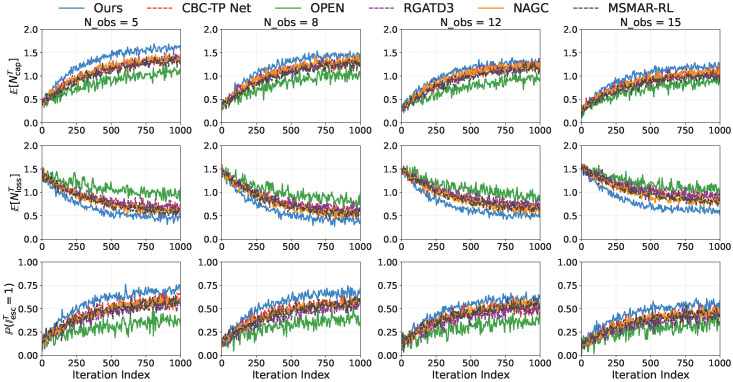
Terminal event statistics under $N_{\mathrm{obs}}=5,8,12,15$: expected captured evaders $E[N_{\mathrm{cap}}^{T}]$, expected lost pursuers $E[N_{\mathrm{loss}}^{T}]$, and escape success probability $P(I_{\mathrm{esc}}^{T}=1)$.

Figure 8 summarizes episodic outcomes. The proposed method improves $E[N_{\mathrm{cap}}^{T}]$ by about 4∼14% over the strongest baseline, reduces $E[N_{\mathrm{loss}}^{T}]$ by about 22∼25%, and increases $P(I_{\mathrm{esc}}^{T}=1)$ by about 13∼20%, with the largest advantage at $N_{\mathrm{obs}}=15$. These differences are also significant under the same seed-level testing protocol. For $E[N_{\mathrm{cap}}^{T}]$, the corrected *p*-values are 0.043,0.031,0.024,0.018 with Cohen’s *d* values of 0.80,0.91,1.00,1.10 across $N_{\mathrm{obs}}=5,8,12,15$. For $E[N_{\mathrm{loss}}^{T}]$, the corrected *p*-values are 0.019,0.014,0.010,0.006 with Cohen’s *d* values of 1.20,1.32,1.45,1.60. For $P(I_{\mathrm{esc}}^{T}=1)$, the corrected *p*-values are 0.028,0.021,0.016,0.010 with Cohen’s *d* values of 0.95,1.08,1.18,1.30. These statistics show that the terminal-event improvements are not only visible in the mean but also practically meaningful, especially for loss reduction and escape success in denser obstacle settings. This indicates more reliable sequencing among approach, encirclement, and disengagement under risk-aware high-level control.

### 5.3. Module-Wise Ablations and Mechanism Verification

All variants in Table 6 are compared under the same evader instance $\pi_{\mathrm{fix}}^{a}$, obstacle generator, and training hyperparameters. To control for randomness from initialization and sampling, each variant is trained with five independent random seeds and evaluated on 500 test episodes after training. Unless otherwise stated, all values in the table are reported as mean ± standard deviation across seeds. Here, Iter@95% is defined as the iteration at which the 100-episode moving-average training curve first reaches 95% of its final plateau value; $T_{\mathrm{step}}$ and $T_{\mathrm{pred}}$ are measured on the same hardware platform after 200 warm-up runs and then averaged over 1000 repeated measurements.

To avoid drawing conclusions from mean differences alone, we further treat random seeds as independent samples and compare the full model against each ablation variant on $\bar{J}\left(\pi \right)$, $P_{\mathrm{cap}}$, $P_{\mathrm{surv}}$, and Viol using Welch’s two-sample *t*-tests. We apply Holm–Bonferroni correction for multiple comparisons and report Cohen’s *d* as the effect size.

First, the generative intent layer mainly improves the structured representation of opponent behavior semantics, rather than merely increasing model capacity. When explicit $\left(z_{t},g_{t}\right)$ generation is removed, G-NoGen shows clear drops in long-horizon return, capture probability, and survival probability. The violation ratio also rises from 1.8% to 2.4%, and convergence becomes slower. This indicates that explicit inference of behavior mode and spatial subgoal benefits not only prediction quality but also downstream policy learning. The result of G-Iso further shows that introducing latent variables alone is not sufficient. The latent prior must also be matched to the current evader state; otherwise, the semantic representation remains less discriminative. The larger degradation of G-Cont and G-CVAE suggests that a generic continuous latent representation or trajectory-level conditional generation cannot fully replace the explicit factorization into behavior mode and geometric subgoal. Hence, the gain of this module does not come from using a generative model per se, but from whether the structured semantic decomposition properly captures the high-level behavioral uncertainty in pursuit–evasion.

Second, the predictor ablations show that the independent length-agnostic predictor is not just a lightweight implementation choice. It is the key component that turns semantic inference into an online-usable predictive capability. P-GenFwd does not cause the largest drop in task-level metrics, but it significantly increases both decision latency and prediction latency, while also slowing convergence. This suggests that directly forwarding the generative model is feasible yet inefficient for real-time prediction in the control loop. By contrast, the distilled length-agnostic predictor preserves predictive capability while substantially reducing online cost. P-NoCons changes average return only slightly, but noticeably increases the violation ratio. This indicates that consistency regularization mainly improves robustness and safety under varying observation-window lengths and partial missing observations, rather than nominal average accuracy. Although P-Attn yields a slightly higher return and capture rate, it also reduces survival, increases violations, and incurs higher latency. Thus, self-attention is not uniformly superior across metrics. In the short-window online pursuit–evasion setting considered here, the GRU-based predictor achieves a better overall balance among accuracy, latency, and safety.

Finally, the decision-layer ablations most clearly reveal the role of belief–risk gating. Compared with the full model, R-Flat nearly doubles the violation ratio and also degrades long-horizon return, capture probability, and survival probability. This suggests that, once the controller is flattened into a single-layer policy, risk information can no longer be adequately absorbed into cooperative decision-making, even if the front-end prediction is retained. In other words, predictive uncertainty must enter high-level decision-making through an appropriate policy structure, rather than being left to a flat controller as an implicit input. R-NoGate partially recovers performance relative to R-Flat, but it remains clearly inferior to the full model. This shows that template mixing alone is helpful but insufficient; without risk-driven gating, a multi-template structure cannot be fully translated into a safety advantage. R-MAD uses the same hierarchical structure but still suffers from lower performance, slower convergence, and more violations. This further indicates that the advantage of the full model does not come from hierarchy alone, but also from the better suitability of MA-SAC for non-stationary and risk-sensitive pursuit–evasion decision-making.

**Figure 9:**
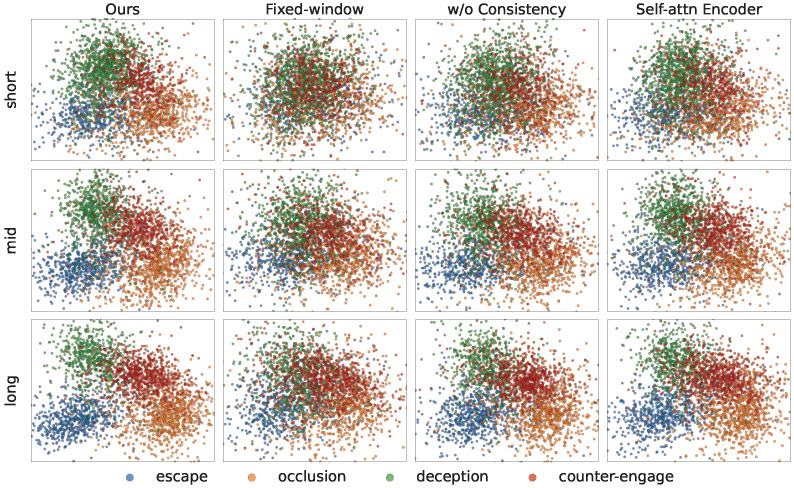
2D visualization of latent-representation separability under observation-window-length shift for ours and three ablations. Colors denote the four evaluation modes.

To examine the stability and separability of behavior-mode representations under varying observation-window lengths, trajectories are collected in an obstacle-rich 2:2 pursuit–evasion environment and evaluated using short, mid, and long windows. Each method projects online latent representations into 2D using the same nonlinear dimensionality-reduction method and colors them according to the four evader behavior modes. The fixed-window approach is trained on a single observation-window length and then tested on others. As shown in Figure 9, our method produces compact and stable clusters with clearer boundaries across all windows, thereby reducing inter-mode overlap. By contrast, fixed-window exhibits pronounced cluster collapse and mixing when the observation-window length changes.

The w/o consistency and self-attention Encoder variants partially preserve cluster structure but show greater drift and overlap. These results suggest that length-agnostic training with distillation and multi-window consistency aligns geometric features across observation-window lengths, thereby reducing time-scale overfitting and semantic mismatch. Removing consistency regularization or merely changing the encoder cannot fully resolve the ambiguity caused by occlusion and non-stationary behavior.

**Figure 10:**
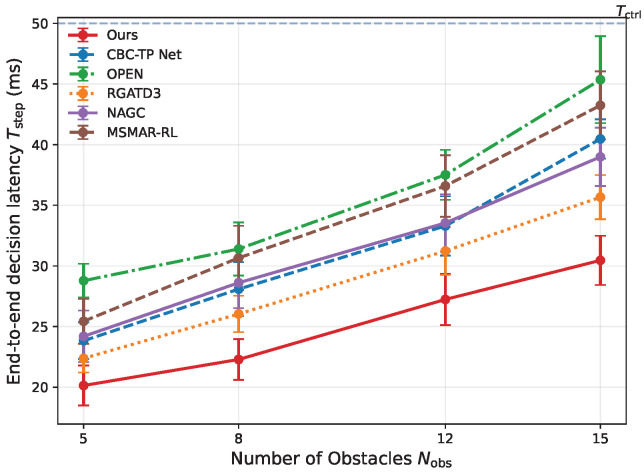
End-to-end decision latency $T_{\mathrm{step}}$ versus obstacle count $N_{\mathrm{obs}}$ (mean ± std over 10 runs). The dashed line denotes $T_{\mathrm{ctrl}}=50\;\mathrm{ms}$.

End-to-end per-step latency $T_{\mathrm{step}}$ under the same hardware and control cycle is reported in Figure 10.

The proposed method shows the mildest latency growth as $N_{\mathrm{obs}}$ increases and remains well below $T_{\mathrm{ctrl}}$. This is mainly because online inference relies on the lightweight predictor rather than generator rollouts, while the high-level module compresses behavior-mode and risk information into low-dimensional gating and fixed-size template mixing.

### 5.4. Sensitivity to Information Conditions and Prediction Structure

#### 5.4.1. Effect of Prediction Horizon

**Figure 11:**
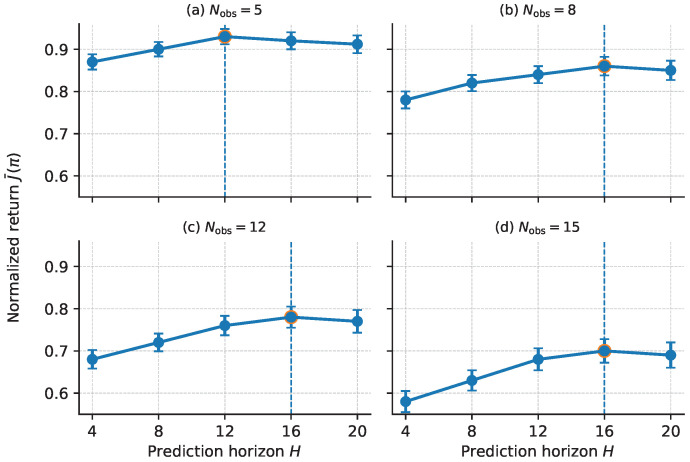
Effect of prediction horizon *H* on $\bar{J}\left(\pi \right)$ under different obstacle settings (mean ± std over 10 runs).

*H* is varied in {4,8,12,16,20} under $N_{\mathrm{obs}}\in \left\{5,8,12,15\right\}$, and the converged $\bar{J}\left(\pi \right)$ is reported in Figure 11. Across environments, $\bar{J}\left(\pi \right)$ first increases with *H* and then decreases slightly because of longer-horizon error accumulation. The best performance typically occurs around H=12 in sparse settings and H=16 in denser settings, indicating that a moderate prediction horizon is important for obstacle-aware coordination.

#### 5.4.2. Observation-Window Length and the Multi-Window Training Paradigm

**Figure 12:**
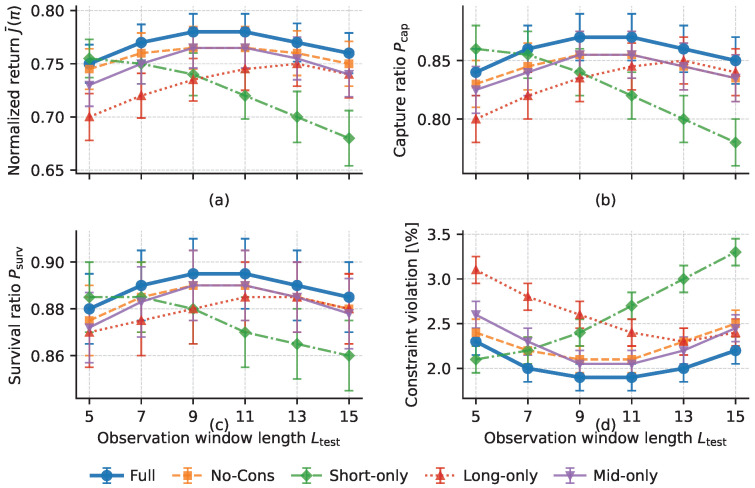
Performance versus test observation-window length $L_{\mathrm{test}}$: (**a**) normalized return $\bar{J}\left(\pi \right)$; (**b**) capture ratio $P_{\mathrm{cap}}$; (**c**) survival ratio $P_{\mathrm{surv}}$; and (**d**) constraint violation ratio (mean ± std over 10 seeds).

Predictor generalization is tested under $L_{\mathrm{test}}\in \left\{5,7,9,11,13,15\right\}$ in the environment with $N_{\mathrm{obs}}=12$, and five training strategies are compared in Figure 12. Multi-window training with consistency regularization is the most stable across $L_{\mathrm{test}}$ and achieves the best overall metrics, whereas single-window strategies overfit to specific observation scales and degrade noticeably when tested at unseen observation-window lengths.

#### 5.4.3. Robustness to Reduced Communication Radius

**Figure 13:**
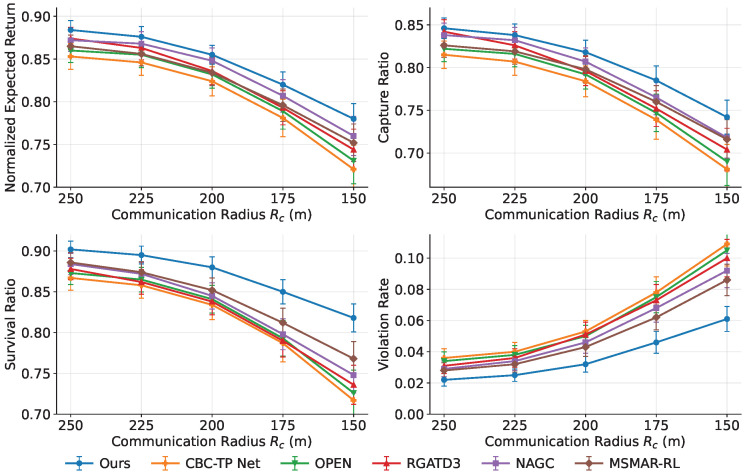
Robustness to reduced communication radius. The communication radius $R_{c}$ is progressively decreased from 250 m to 150 m, and four metrics are reported: normalized expected return, capture ratio, survival ratio, and violation rate.

To further examine robustness under tighter geometry-induced communication constraints, we evaluate all methods by progressively reducing the communication radius during testing while keeping the remaining environment settings and training protocol unchanged. Specifically, the communication radius $R_{c}$ is decreased from its nominal value of 250 m to 225 m, 200 m, 175 m, and 150 m, and the normalized expected return, capture ratio, survival ratio, and violation rate are recorded under each setting, as shown in Figure 13.

As the communication radius decreases, all methods exhibit lower normalized expected return, capture ratio, and survival ratio, together with a steadily increasing violation rate, indicating that stricter communication constraints substantially impair information sharing and coordinated decision-making. Nevertheless, the proposed method consistently achieves the best performance across all communication-radius settings and degrades more gracefully than the baselines. Under the most restrictive condition, $R_{c}=150\;m$, the proposed method still attains a normalized expected return of 0.780, a capture ratio of 0.742, and a survival ratio of 0.818, while keeping the violation rate to 0.061. Relative to the strongest baseline at this setting, it preserves advantages of 0.020, 0.024, and 0.050 on these three positive metrics, respectively, and further reduces the violation rate by 0.025. These results indicate that the proposed framework remains more robust in both cooperative pursuit effectiveness and risk control when communication becomes more severely constrained.

### 5.5. Parameter Sensitivity of the Efficiency–Safety Trade-Off

#### 5.5.1. Effect of $\kappa_{\mathrm{eng}}$

**Figure 14:**
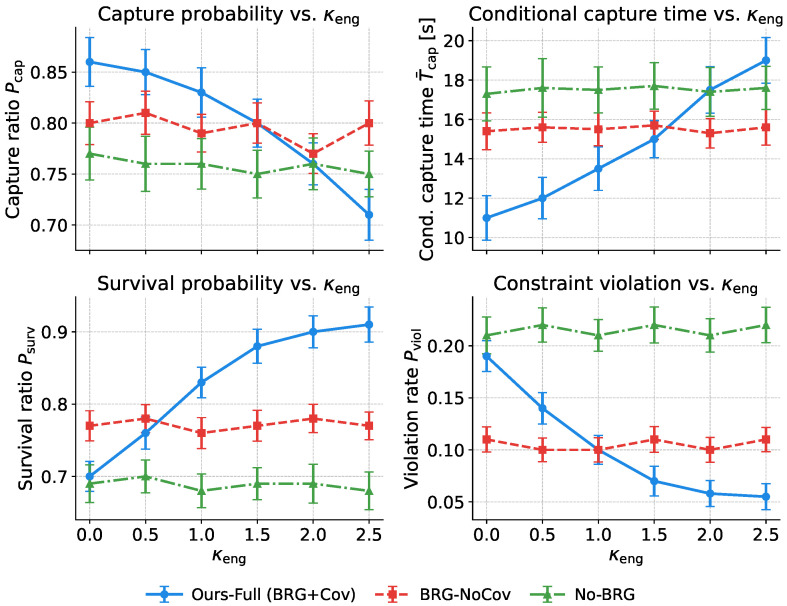
Sensitivity to $\kappa_{\mathrm{eng}}$: BRG+Cov (full), BRG-NoCov, and No-BRG. Panels report $P_{\mathrm{cap}}$, ${\bar{T}}_{\mathrm{cap}}$, $P_{\mathrm{surv}}$, and $P_{\mathrm{viol}}$.

With trained policies fixed, $\kappa_{\mathrm{eng}}\in \left\{0.0,0.5,1.0,1.5,2.0,2.5\right\}$ is varied, and Ours-Full (BRG+Cov) is compared with BRG-NoCov and No-BRG in Figure 14. Ours-Full exhibits a clear efficiency–safety trade-off: increasing $\kappa_{\mathrm{eng}}$ improves survivability and reduces violations but gradually lowers capture efficiency and increases capture time. BRG-NoCov shows weaker sensitivity, whereas No-BRG performs worst across all $\kappa_{\mathrm{eng}}$, indicating that covariance-aware inflation and belief–risk gating are both important for controllable policy switching. In our setting, $\kappa_{\mathrm{eng}}\in \left[1.0,1.5\right]$ provides a practical compromise.

#### 5.5.2. Effects of $\delta_{\mathrm{th}}$ and $\delta_{\mathrm{safe}}$

**Figure 15:**
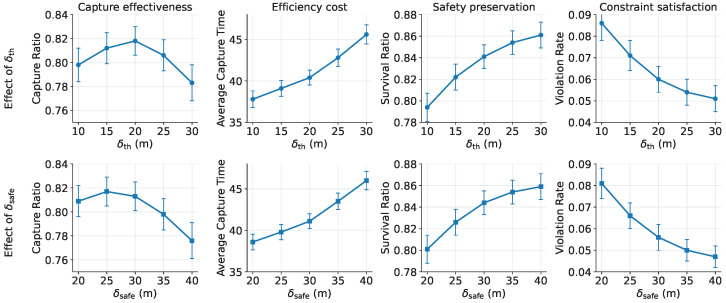
Sensitivity of the efficiency–safety trade-off to additional core parameters. The top row varies the threat-trigger threshold $\delta_{\mathrm{th}}$, and the bottom row varies the desired minimum safety distance $\delta_{\mathrm{safe}}$. Four metrics are reported for each setting: capture ratio, average capture time, survival ratio, and violation rate.

To further examine how the efficiency–safety trade-off depends on key design parameters, we evaluate the effects of the threat-trigger threshold $\delta_{\mathrm{th}}$ and the desired minimum safety distance $\delta_{\mathrm{safe}}$ while keeping the remaining environment settings and training protocol unchanged. Specifically, $\delta_{\mathrm{th}}$ is varied over {10,15,20,25,30}m and $\delta_{\mathrm{safe}}$ over {20,25,30,35,40}m. For each setting, the capture ratio, average capture time, survival ratio, and violation rate are recorded, as shown in Figure 15.

As shown in Figure 15, both parameter groups exhibit a clear efficiency–safety trade-off. As $\delta_{\mathrm{th}}$ increases from 10m to 30m, the average capture time rises steadily from 37.8s to 45.6s, while the survival ratio improves from 0.794 to 0.861 and the violation rate decreases from 0.086 to 0.051. Meanwhile, the capture ratio first increases and then declines, reaching its maximum value of 0.818 at $\delta_{\mathrm{th}}=20\;m$. A similar pattern is observed for $\delta_{\mathrm{safe}}$. As $\delta_{\mathrm{safe}}$ increases from 20m to 40m, the average capture time rises from 38.6s to 46.0s, the survival ratio increases from 0.801 to 0.859, and the violation rate decreases from 0.081 to 0.047, whereas the capture ratio peaks at 0.817 when $\delta_{\mathrm{safe}}=25\;m$ and then gradually declines to 0.776. These results indicate that overly small thresholds or safety margins expose the team to higher violation risk, whereas overly conservative settings sacrifice pursuit efficiency. Overall, $\delta_{\mathrm{th}}=20\;m$ and $\delta_{\mathrm{safe}}=25–30\;m$ provide a well-balanced operating region.

### 5.6. Scalability to Larger Team Sizes

**Figure 16:**
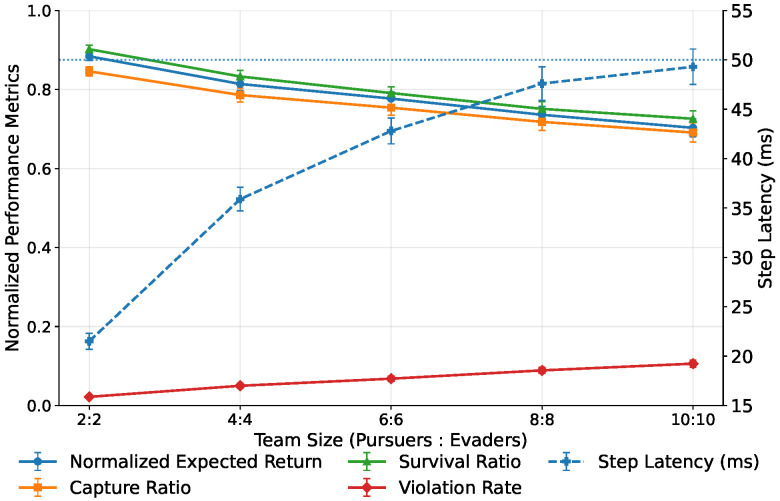
Scalability profile of the proposed method under symmetrically increasing team sizes. The numbers of pursuers and evaders are jointly expanded from 2:2 to 10:10. The figure reports the normalized expected return, capture ratio, survival ratio, violation rate, and step latency.

To assess the applicability of the proposed method to larger-scale cooperative pursuit–evasion tasks, we further examine how mission effectiveness, safety, and real-time performance evolve as the team size increases symmetrically. Specifically, the numbers of pursuers and evaders are expanded jointly from 2:2 to 4:4, 6:6, 8:8, and 10:10. For each scale, the normalized expected return, capture ratio, survival ratio, violation rate, and step latency are recorded, as shown in Figure 16.

As the pursuit–evasion scale increases, the normalized expected return, capture ratio, and survival ratio all decrease gradually, whereas the violation rate and step latency increase steadily, indicating that larger teams impose substantially greater demands on coordination, information exchange, and safety preservation. Nevertheless, even at the largest tested scale of 10:10, the proposed method still maintains a normalized expected return of approximately 0.70, a capture ratio of about 0.69, and a survival ratio of about 0.73, while keeping the violation rate near 0.11. At the same time, although the step latency increases to about 49ms, it remains within the 50ms control cycle. These results suggest that the proposed framework degrades in a stable and predictable manner as the team size grows, while still preserving acceptable mission performance, safety, and online real-time capability.

### 5.7. Representative Episodes and Robustness to Obstacle Layouts

**Figure 17:**
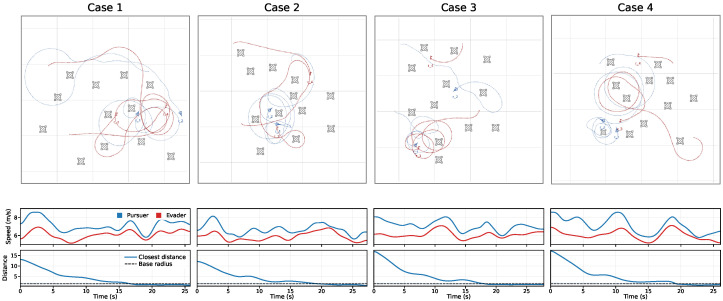
Representative capture episodes across four maps, in which both pursuers survive and both evaders are captured. **Top**: trajectories, where blue dashed curves denote pursuer trajectories, red dashed curves denote evader trajectories, arrowheads indicate the motion direction, and gray hatched squares denote obstacles. **Middle**: average speeds of pursuers and evaders. **Bottom**: closest inter-team distance over time, with the black dashed line denoting the base capture radius, showing progressive distance compression until capture.

Representative episodes under randomized obstacle layouts are visualized to assess behavioral consistency and robustness. Figure 17 shows episodes in which both pursuers survive and both evaders are captured. Across maps, one pursuer maintains pressure while the other detours to complete the encirclement, with detours concentrated near corners and narrow passages to preserve obstacle and formation margins. The speed traces show that the pursuers maintain a higher pace than the evaders and exhibit short bursts near closure moments, consistent with a strategy that first secures positional advantage and then tightens the enclosure. The closest distance decreases progressively and eventually stabilizes around the capture threshold, indicating reliable distance compression within safety margins.

**Figure 18:**
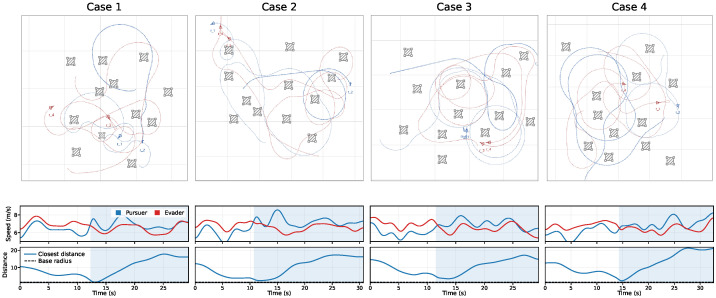
Representative episodes across four maps in which one pursuer is lost while the remaining pursuer survives. **Top**: trajectories, where blue dashed curves denote pursuer trajectories, red dashed curves denote evader trajectories, arrowheads indicate the motion direction, and gray hatched squares denote obstacles. **Middle**: average speeds of pursuers and evaders. **Bottom**: closest inter-team distance over time, with the black dashed line denoting the base capture radius; the shaded interval marks the retreat phase, during which the distance is restored to a safe margin.

Figure 18 shows episodes in which one pursuer is lost while the other survives. Under unfavorable local geometry, the remaining pursuer enters a retreat mode and uses obstacle shielding to restore separation, reflecting a deliberate risk-aware trade-off between efficiency and safety. This is evidenced by a retreat interval during which the speed profile becomes more conservative and the closest distance rebounds after brief near-threshold contact before remaining above a safe margin. These results confirm that the risk-aware safety mechanism actively adjusts the engagement behavior.

## 6. Conclusions

This study investigates cooperative multi-UAV pursuit–evasion under intermittent observability, varying observation-window lengths, and non-stationary evader behavior modes in obstacle-rich environments. A three-stage closed-loop framework was developed to transform short observation windows into structured behavior-mode and subgoal representations, distill this inference capability into a length-agnostic online predictor with uncertainty quantification, and learn a belief–risk-gated hierarchical MA-SAC policy with an explicit safety projection layer. Experiments across obstacle densities, randomized layouts, reduced communication radii, and varying observation-window lengths demonstrated consistent improvements in return, capture performance, survivability, and constraint satisfaction, while maintaining real-time feasibility for online deployment.

Although all experiments in this study were conducted against the fixed evader policy instance $\pi_{\mathrm{fix}}^{a}$, this evaluation setting was adopted to isolate pursuer-side learning performance under consistent adversarial conditions. The proposed framework explicitly decouples opponent-intent inference, uncertainty-aware prediction, and belief–risk-gated decision-making, allowing the pursuer team to adjust its cooperative behavior when the evader’s behavior mode changes or when predictive uncertainty increases. This decoupled structure therefore suggests potential applicability to more adaptive or learning-based opponents.

Moreover, the proposed framework is not inherently restricted to planar pursuit–evasion with static obstacles. By extending the state representation, observation model, and safety-projection mechanism, the same pipeline can in principle be generalized to 3D environments and dynamic-obstacle scenarios, although the associated real-time computational burden and scalability still require dedicated validation. Future work will further extend the framework to larger swarms under communication constraints, with emphasis on decentralized belief updating, scalable risk assessment, and evaluation against more adaptive adversarial policies.

## Figures and Tables

**Table 1:** Notation summary for the main symbols in the system model.

Symbol	Meaning
$N^{f},N^{a}$	Index sets of pursuers and evaders
$x_{k}\left(t\right)$	State of UAV *k*, composed of position and velocity
$p_{k}\left(t\right),\;v_{k}\left(t\right)$	Position and velocity of UAV *k*
$u_{k}\left(t\right)$	Acceleration control input of UAV *k*
$X_{t}$	Global physical state of all UAVs at step *t*
$G_{t}^{\mathrm{comm}}$	Time-varying communication graph of the pursuer team
$N_{i}^{\mathrm{comm}}\left(t\right)$	Communication-neighbor set of pursuer *i*
$N_{i}^{\mathrm{vis}}\left(t\right)$	Set of evaders visible to pursuer *i*
$o_{i}^{t}$	Local observation of pursuer *i* at step *t*
$z_{t}$	Evader behavior mode
$g_{t}$	Evader short-horizon subgoal
$\eta_{t}=\left(z_{t},g_{t}\right)$	Latent intent variable of the evader
$S_{t}=\left(X_{t},z_{t},g_{t}\right)$	Augmented state at step *t*

**Table 2:** Implementation and training hyperparameters of LaTP.

Item	Value
Encoder	2-layer GRU, hidden size 128
Prediction heads	4 linear heads: mode, goal mean, goal covariance, trajectory
Activation	ReLU in shared layers; linear output heads; exponential map for covariance
Optimizer	Adam
Learning rate	$3\times 10^{-4}$
Batch size	256
Weight decay	$1\times 10^{-5}$
Gradient clipping	Global norm clipping at 10.0
Loss weights	$\lambda_{\mathrm{mode}}=1.0,\;\lambda_{\mathrm{goal}}=2.0,\;\lambda_{\mathrm{traj}}=1.0$
Consistency/uncertainty weights	$\beta_{\mathrm{cons}}=0.5,\;\lambda_{\Sigma }=0.1,\;\lambda_{\Sigma }^{\mathrm{cons}}=0.05$
Training budget	100 epochs
Stopping rule	Best validation loss; early stopping patience = 10

**Table 3:** Implementation and training hyperparameters of BR-HMASAC.

Item	Value
High-level gate network	MLP with 2 hidden layers of 128 units
Template-weight network	MLP with 2 hidden layers of 128 units
Low-level Gaussian actor	MLP with 2 hidden layers of 256 units
Centralized critic	Twin Q-networks, each with 2 hidden layers of 256 units
Activation	ReLU
Actor log-std clipping	[−5,2]
Optimizer	Adam
Learning rates	actor: $3\times 10^{-4}$, critic: $3\times 10^{-4}$, temperature: $1\times 10^{-4}$
Replay buffer	$10^{6}$ transitions
Batch size	256
Discount factor	γ=0.99
Soft target update	$\tau =5\times 10^{-3}$
Entropy coefficient	automatic tuning; initial α=0.2
Target entropy	−2 per agent
Warm-up steps	5000
Update frequency	1 gradient update per environment step
Training budget	1000 episodes
Model selection	best checkpoint by moving-average return over the last 100 episodes

**Table 4:** Task environment and UAV parameter settings.

Item	Symbol	Value
Workspace	Ω	$\left[0,1000\right]\times \left[0,1000\right]\;m^{2}$
Discrete time step	Δt	0.1s
Max steps per episode	$T_{\max}$	300 (about 30s)
No. of pursuers	$N_{f}$	2
No. of evaders	$N_{a}$	2
No. of obstacles	$N_{\mathrm{obs}}$	{5,8,12,15}
Min initial pursuer–evader distance	–	$3R_{\mathrm{eng}}$
Max speed	$v^{\max}$	40m/s
Max acceleration	$a^{\max}$	$15\;m/s^{2}$
Initial velocity direction	–	Uniform over [0,2π)
Desired minimum safety distance	$\delta_{\mathrm{safe}}$	30m
Pursuer communication radius	$R_{c}$	250m
Sensor range	$R_{s}$	300m
Field of view	$\varphi_{s}$	$120^{\circ }$
Engagement radius	$R_{\mathrm{eng}}$	80m
Engagement angle	$\varphi_{\mathrm{eng}}$	$60^{\circ }$
Min dwell steps for engagement	$\tau_{\mathrm{eng}}$	5

**Table 5:** Prediction, reward, and index parameter settings.

Item	Symbol	Value
Prediction horizon	*H*	16
Min observation-window length	$L_{\min}$	5
Max observation-window length	$L_{\max}$	20
Mid observation-window length	$L_{\mathrm{mid}}$	10
Threat buffer threshold	$\delta_{\mathrm{th}}$	20m
Engagement-radius inflation coefficient	$\kappa_{\mathrm{eng}}$	1.0
Capture reward weight	$w_{\mathrm{cap}}$	1.0
Survival reward weight	$w_{\mathrm{surv}}$	0.8
Safety reward weight	$w_{\mathrm{safe}}$	0.6
Control-smoothness reward weight	$w_{\mathrm{ctrl}}$	0.2
Terminal reward (per capture)	$R_{\mathrm{cap}}$	+10.0
Terminal reward (successful escape)	$R_{\mathrm{esc}}$	+8.0
Terminal penalty (per pursuer loss)	$R_{\mathrm{loss}}$	5.0
Discount factor	γ	0.99
Safety index parameter	$\alpha_{\mathrm{safe}}$	0.10
Capture index parameter	$\alpha_{\mathrm{cap}}$	0.05
Control index parameter	$\alpha_{\mathrm{ctrl}}$	0.01

**Table 6:** Module-wise ablation results.

Variant	Module Combo.	$\bar{J}\left(\pi \right)\;↑$	$P_{\mathrm{cap}}\;↑$	$P_{\mathrm{surv}}\;↑$	Viol[%]↓	Iter@95%↓	$T_{\mathrm{step}}\left[\mathrm{ms}\right]\;↓$	$T_{\mathrm{pred}}\left[\mathrm{ms}\right]\;↓$
Full	$\left(G_{1},P_{1},R_{1}\right)$	0.81±0.02	0.87±0.05	0.89±0.04	1.8±0.68	245±37	3.91±0.22	1.43±0.18
G-NoGen	$\left(G_{0},P_{1},R_{1}\right)$	0.76±0.02	0.82±0.03	0.85±0.04	2.4±0.66	268±33	3.77±0.19	1.38±0.10
G-Iso	$\left(G_{2},P_{1},R_{1}\right)$	0.78±0.03	0.84±0.05	0.86±0.03	2.1±0.52	259±36	3.88±0.12	1.52±0.20
G-Cont	$\left(G_{3},P_{1},R_{1}\right)$	0.73±0.04	0.79±0.03	0.83±0.04	2.8±0.80	281±15	4.03±0.22	1.57±0.23
G-CVAE	$\left(G_{4},P_{1},R_{1}\right)$	0.72±0.04	0.78±0.04	0.82±0.03	3.0±0.54	292±22	3.93±0.21	1.48±0.16
P-GenFwd	$\left(G_{1},P_{0},R_{1}\right)$	0.79±0.04	0.86±0.05	0.88±0.03	2.0±0.59	276±19	5.07±0.21	2.83±0.15
P-NoCons	$\left(G_{1},P_{2},R_{1}\right)$	0.80±0.04	0.86±0.04	0.88±0.04	2.5±0.68	252±23	3.84±0.30	1.37±0.12
P-Attn	$\left(G_{1},P_{3},R_{1}\right)$	0.82±0.04	0.88±0.04	0.87±0.04	2.2±0.63	238±26	4.36±0.20	1.92±0.09
R-Flat	$\left(G_{1},P_{1},R_{0}\right)$	0.74±0.02	0.80±0.05	0.84±0.04	3.4±0.67	261±37	3.18±0.25	1.28±0.10
R-NoGate	$\left(G_{1},P_{1},R_{2}\right)$	0.77±0.03	0.83±0.04	0.87±0.03	2.9±0.83	254±37	3.73±0.29	1.42±0.18
R-MAD	$\left(G_{1},P_{1},R_{3}\right)$	0.78±0.04	0.84±0.03	0.86±0.03	2.3±0.53	273±31	4.02±0.21	1.39±0.11

↑ denotes that higher values are preferred, whereas ↓ denotes that lower values are preferred. $G_{0}$: no generative model; $G_{1}$: explicit $\left(z_{t},g_{t}\right)$ generation with prior conditioned on $X_{t}^{a}$; $G_{2}$: isotropic fixed prior; $G_{3}$: single continuous latent variable; $G_{4}$: conditional VAE trajectory generator without explicit $\left(z_{t},g_{t}\right)$. $P_{0}$: no independent predictor, directly forwarding the generative model; $P_{1}$: length-agnostic predictor with distillation and multi-window consistency; $P_{2}$: removing consistency regularization; $P_{3}$: replacing the GRU encoder with self-attention. $R_{0}$: flat MA-SAC; $R_{1}$: hierarchical MA-SAC with belief–risk gating and template mixing; $R_{2}$: removing gating while keeping template mixing only; $R_{3}$: using MADDPG under the same hierarchical structure.

## Data Availability

Dataset available on request from the authors. The raw data supporting the conclusions of this article will be made available by the authors on request.
